# Identification of a multi-cancer gene expression biomarker for cancer clinical outcomes using a network-based algorithm

**DOI:** 10.1038/srep11966

**Published:** 2015-07-23

**Authors:** Emmanuel Martinez-Ledesma, Roeland G.W. Verhaak, Victor Treviño

**Affiliations:** 1 Grupo de Enfoque e Investigación en Bioinformática, Departamento de Investigación e Innovación, Escuela Nacional de Medicina, Tecnológico de Monterrey, Monterrey, Nuevo León 64849, México; 2 Department of Genomic Medicine, The University of Texas MD Anderson Cancer Center, Houston, Texas 77030, USA; 3 Department of Bioinformatics and Computational Biology, The University of Texas MD Anderson Cancer Center, Houston, Texas 77030, USA

## Abstract

Cancer types are commonly classified by histopathology and more recently through molecular characteristics such as gene expression, mutations, copy number variations, and epigenetic alterations. These molecular characterizations have led to the proposal of prognostic biomarkers for many cancer types. Nevertheless, most of these biomarkers have been proposed for a specific cancer type or even specific subtypes. Although more challenging, it is useful to identify biomarkers that can be applied for multiple types of cancer. Here, we have used a network-based exploration approach to identify a multi-cancer gene expression biomarker highly connected by ESR1, PRKACA, LRP1, JUN and SMAD2 that can be predictive of clinical outcome in 12 types of cancer from The Cancer Genome Atlas (TCGA) repository. The gene signature of this biomarker is highly supported by cancer literature, biological terms, and prognostic power in other cancer types. Additionally, the signature does not seem to be highly associated with specific mutations or copy number alterations. Comparisons with cancer-type specific and other multi-cancer biomarkers in TCGA and other datasets showed that the performance of the proposed multi-cancer biomarker is superior, making the proposed approach and multi-cancer biomarker potentially useful in research and clinical settings.

Cancer is typically classified by tissue-specific scores such as the Gleason score in prostate cancer^1^, the Dukes or Astler-Coller in colon cancer^2^, or Figo in cervical cancer^3^. These have been generalized by TNM staging^4^. More recently, high-throughput technologies have generated unprecedented molecular characterizations of cancer types, such as the genomic portrayals provided by The Cancer Genome Atlas (TCGA) research network^5^,^6^,^7^. Several cancer types have been divided into subtypes using TCGA data about gene expression^8^, mutations^9^, copy number alterations^10^, microRNA expression^11^, pseudogenes^12^, or even biological processes such as inflammation^13^. Nevertheless, specific subtypes across cancer types seem to share common gene expression properties such as correlations^14^,^15^, stromal and immune signatures^16^, or mesenchymal signatures^17^. Clinically, better or alternative methods to identify cancer risk groups are always needed.

Although point mutations and chromosomal alterations are currently the subject of active research, a large reservoir of public research has been devoted to the study of gene expression^18^,^19^, which is the most broadly studied class of molecular data for cancer so far. More importantly, gene expression is a consequence of cumulative genetic and epigenetic alterations. With the goal of clinically stratifying samples into risk groups, several gene expression biomarkers have been proposed for a large variety of cancer types^20^,^21^,^22^,^23^,^24^,^25^.

However, most biomarkers have been identified and designed for a specific type of cancer. Moreover, some biomarkers can be applied only to specific subtypes; for example, biomarkers exist specifically for grade 2 in colon cancer^26^ and estrogen receptor-positive and lymph node-negative in breast cancer^27^. Many proposed gene signatures may not even be considered for clinical use because they could not be reliably validated in other cohorts^28^. In some cases, a lack of agreement has also been reported among gene expression signatures obtained for the same type of cancer^29^. In addition, it was recently shown that biomarkers identified for only one cancer type perform modestly or poorly even when clinical data are considered^30^.

Gene expression biomarkers that can be applied for a broad range of cancers could be highly useful in research and clinical settings. In clinics, such biomarkers may serve as a standard assessment for facilitating the interpretation and broad application of laboratory test results, simplifying laboratory protocols, and reducing costs. In research, these biomarkers may help to elucidate broadly observed biological mechanisms and possible drug targets. Nevertheless, gene expression biomarkers that can be applied to more than one cancer type are scarce. Most studies exploit specific properties to identify multi-cancer signatures. For example, signatures have been identified from metastasis-specific solid tumors^31^, or found to be associated with chromosomal instability^32^, therapy-failures^33^, proliferation signatures^34^, subsequent cancers^35^, and embryonic stem-cell like gene expression^36^.

Distinct algorithms and strategies have been used to identify biomarkers for more than 10 years. These methods include variable selection by shrinkage^37^,^38^, penalization^39^,^40^, clustering^41^, differential expression^42^,^43^, or simply by selection of the top-ranked genes using a univariate Cox score^21^, among many others^44^. Most of these methods nevertheless do not consider *a priori* biological information to identify gene signatures. The use of biological information adds a layer of validation and prioritization^45^ that can be exploited for biomarker discovery. Common approaches that consider biological information use networks such as protein-protein interactions (PPI) or gene ontologies, which drives the search for modules or terms that could function as gene signatures. For example, a set of significant subnetwork biomarkers to classify breast cancer metastasis was identified by performing a greedy search starting from seed genes and then adding neighbor genes^46^. These subnetworks were then compared with a null distribution of random subnetworks. Similarly, this algorithm was adapted for a web server that provides network-based biomarkers for survival data^47^. A network module-based approach applied a Markov clustering algorithm to the correlation of the PPI matrix identifying modules associated with patient survival^48^. An algorithm similar to that used by Google for ranking web pages has been proposed to order genes according to their association with survival outcomes^49^. Modularity has also been suggested as an indicator of breast cancer prognosis as determined by an algorithm to find intramodular highly co-expressed and highly interconnected “hub” genes and intermodular hub genes with low co-expression^50^. Moreover, gene ontology has also been used to identify metastasis network modules combining highly predictive gene ontology sets^51^. To the best of our knowledge, these network-based approaches have not been tailored to produce network-based multi-cancer biomarkers.

Here we describe a network-based approach that explores, in parallel, gene-to-gene connections in multiple cancer datasets while maximizing the overall association of the subnetwork with clinical outcomes. We implemented this network-based algorithm using, as a proof of concept, the Human Protein Reference Database (HPRD)^52^, 12 TCGA cancer types^53^, and a composite Cox-based model^54^. In these training datasets, the results showed that a gene signature of 41 genes was capable of predicting risk groups across cancer types with high precision. Analysis of a large collection of clinical outcome cancer datasets that included cancers types reported by several authors and many cancer types that were not included in the training datasets validated these results. The predictive power of the biomarker was higher than that of clinical information alone and improved when combined. Our results suggest that it is possible to identify general, compact, and biologically driven gene expression biomarkers for multiple cancer types.

## Material and Methods

### Datasets

We used data from 12 cancer types that belong to the TCGA pan-cancer project repository^53^ accessed in January 2013, compiled in 11 datasets (http://nature.com/tcga). Detailed lists of datasets, genes and samples used are shown in Table 1 and Supplementary Table 1. Level 3 data were used. Only gene symbols present in all cancer types were used. Microarray data (Agilent and Affymetrix) were transformed using quantile normalization. RNA-Seq data were Log2 transformed and quantile normalized.

### Biological networks

We used the protein-protein interaction (PPI) network from the Human Protein Reference Database (HPRD)^52^ accessed in March 2013. The network covered 9,465 genes and 37,080 interactions. Only genes found in both the TCGA datasets and the network were used.

### Performance of network modules

We used gene expression values from each cancer type fitting a Cox model to measure the level of association of a given gene signature. For biomarkers specific to a cancer-type, the negative logarithm of the log-rank test (NLLRT) was used to assess and drive the network-based search. For the multi-cancer biomarker, we used the NLLRT of a reference cancer-type minus the range of the NLLRT from the remaining cancer-types. Subtracting the range of values gave preference to less variable signatures, helping to avoid over-fitting to specific cancer types. We used GBM as the reference cancer-type because its performance was the lowest across the cancer type-specific runs. Nevertheless, we also used other cancer types as reference and compared the results. To assess the overall performance of the prediction after biomarker identification, we used the concordance index (C-index) measure, which is similar to the area under the receiving operating curve^55^. C-index values close to 0.5 are referred to as random risk predictions whereas C-index values close to 1 are interpreted as nearly perfect risk predictions. To represent the performance of biomarkers graphically, we split the samples by the median of the prognostic index to designate low- and high- risk groups. The prognostic index is the linear component of the exponential function in the Cox model.

### Network clinical association (NCA) algorithm

**Figure 1:**
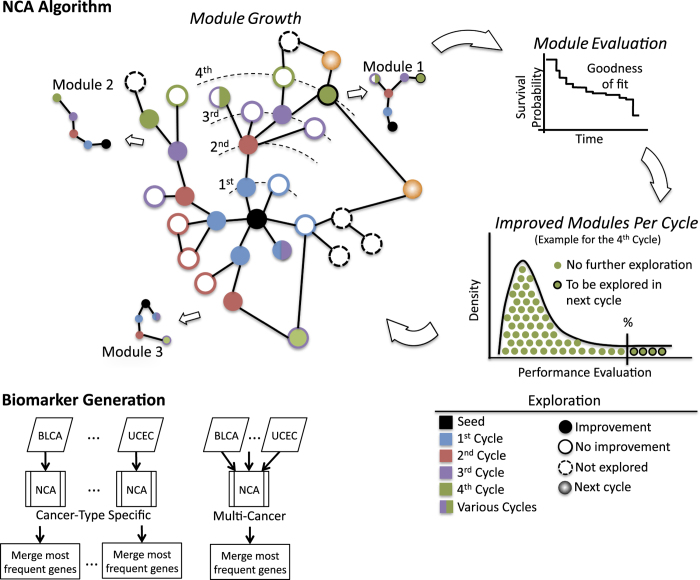
Schematic representation of the network clinical association algorithm (NCA). Starting from a single seed gene (black), the first cycle generates modules that include the seed gene and each of the connected genes (blue). The 6 modules of 2 genes are then evaluated by their goodness of fit in a Cox survival model. Only those grown modules that improve (filled blue circles) the evaluation are considered for the next grow cycle. Only a proportion of the best improved modules are further explored in the next cycle (represented by a percentage of the distribution of all modules, shown in green, evaluated in the 4th cycle). This procedure continues until no improvement is observed. The NCA algorithm was run for each cancer type and for all cancer datasets (multi-NCA).

Figure 1 shows a graphical representation of the NCA algorithm. The algorithm proceeds in cycles, starting with the determination of the performance of an isolated gene (a seed gene) across all datasets. Then, a module growth cycle is performed, in which all connected genes are explored, one gene at a time, generating as many grown modules as connections. In the exploration, the performance of the module is evaluated using the NLLRT value described above. Afterwards, only the top 5% of the modules whose NLLRT value improved after the addition of new connections are considered for the next growth cycle. The procedure continues until no improvement is observed. The algorithm starts by using each gene as a seed. This algorithm functions as a type of hill-climbing algorithm. Scripts or executables of this algorithm are available from the corresponding author.

### Validation analysis

To determine the significance of the C-index values, we generated a null distribution composed of 10,000 random models of 41 genes for the TCGA datasets we used. To assess the C-index prediction of the biomarkers in datasets other than TCGA, we used SurvExpress^56^, which provides evaluations of gene lists across cancer types. For this, we used normalized datasets that included overall survival times (without considering recurrence, metastases, or relapse) and only those studies containing more than 30 samples. For replicated genes, we selected the highest expressed probe. Analyses were performed in R (http://cran.r-project.org/). For biological validation, we used MSigDB and DAVID^57^,^58^ to determine which biological terms were associated with the biomarker gene lists. We also compared the C-index values of our multi-cancer biomarker with those of other multi-cancer biomarkers reported in the literature. For model comparisons including clinical features (e. g. cancer staging), we used the “other factors” option in SurvExpress.

## Results

### Identification of biomarkers for specific cancer types

**Figure 2:**
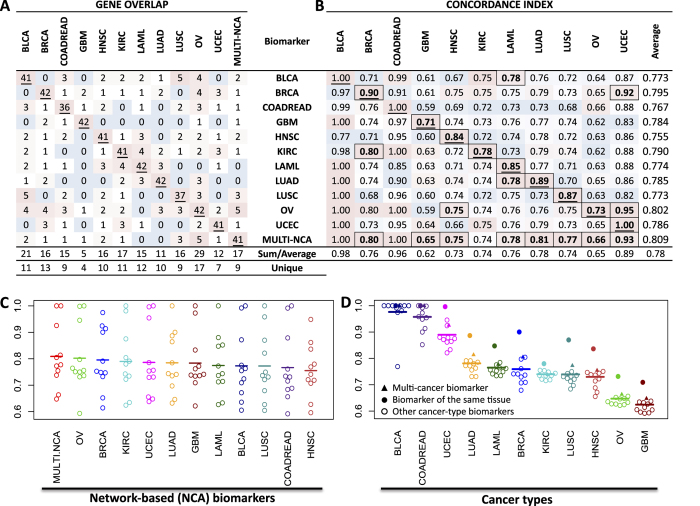
Comparison of biomarkers generated by the network clinical association (NCA) algorithm. Panel **A** shows the number of genes that were included in any two biomarkers. Underlined numbers represent the number of genes per biomarker. Red indicates high overlaps and blue indicates no overlap. The “Sum” row shows the total number of overlaps with other biomarkers while the “Unique” row shows the number of unique genes that overlap. Panel **B** shows the C-index evaluation of NCA biomarkers (rows) across cancer datasets (columns). Underlined numbers represent the biomarkers evaluated within the cancer dataset. Red indicates high values within the cancer dataset (column) and blue indicates low values. Boldface and framed values represent significant predictions using 10,000 random models of the same length. The “Average” row shows the average C-index per cancer type and the “Average” column shows the mean C-index per biomarker. Panel **C** shows the NCA biomarkers (horizontal) evaluated in all datasets using C-index (vertical axis). The mean is shown as a horizontal line. Panel **D** shows cancer types (horizontal) evaluated with all biomarkers using C-index (vertical axis).

We first executed the NCA algorithm (Fig. 1) for each of the 11 cancer datasets. We focused on the network modules with the highest performance value. The results shown in Table 2 suggest that, in general, several network modules existed for each cancer type, from 84 for LUSC to 10,303 for BLCA. Most cancer types generated modules with about 9 genes, ranging in size from 4 for KIRC to 14 for LUSC. To generate a biomarker that is representative of all modules for a specific cancer type, we used the genes that most frequently occurred in modules (around 41 genes for comparisons with the multi-cancer biomarker). The list of genes obtained is provided in Supplementary Table 2. Comparisons of the genes used for these biomarkers across cancer types showed that the pairwise gene overlap was low (ranging from 0 to 5, see Fig. 2A). Although the specific genes used for each biomarker were clearly different, indicating that the biomarkers are cancer type-specific, the prediction across cancer types was surprisingly satisfactory; the average C-index values were higher than 0.75 (Fig. 2B and Supplementary Table 3). Almost all cancer type-specific biomarkers showed C-index values higher than 0.70 for about 8 cancer types (Fig. 2C). We observed consistent C-index values within each cancer type almost irrespective of the network-based biomarker (Fig. 2D). For instance, all biomarkers had a C-index value about 0.97 for BLCA and 0.95 for COADREAD but about 0.65 for OV and 0.62 for GBM. Nevertheless, a random signature analysis indicated that only 14 of the 121 C-index values (11.5%) were significant, mainly those of cancer type-specific biomarkers within the same cancer type dataset (excluding BLCA and COADREAD, see Fig. 2A and Supplementary Table 3).

### Identification of a multi-cancer biomarker

To generate a broadly predictive biomarker, we used the NCA algorithm and considered the 11 datasets in the same run. We estimated a composite performance score based on the individual performance of all cancer types. We maximized the overall performance by taking the NLLRT of a reference cancer type (glioblastoma) and subtracting the range of NLLRT values of the other cancer types. In this way, genes generating large deviances for specific cancer types were avoided in favor of the inclusion of genes that improved the prediction in many cancer types. Two very similar modules consisting of 44 genes were identified (Table 2). Only 6 genes were not present in both modules (JDP2, KIF5B, NTRK3, MMP13, TGFB1, and TGFBRAP1). Therefore, we used the genes present in both modules as the overall multi-cancer biomarker.

**Figure 3:**
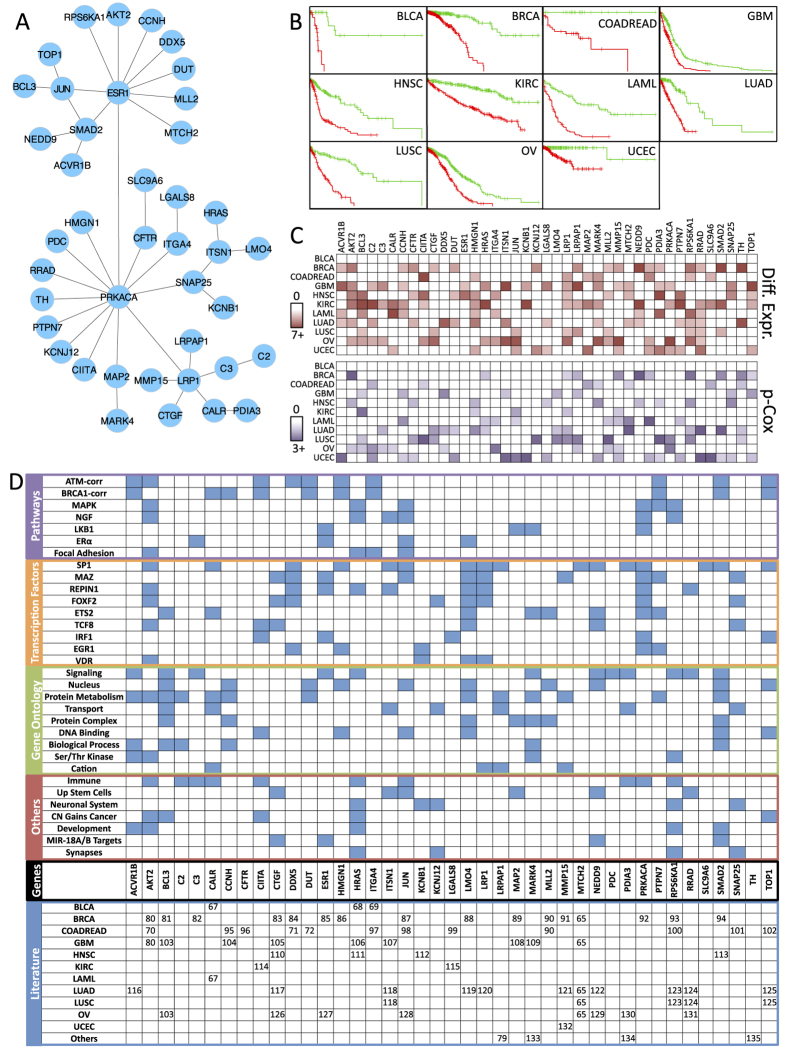
The multi-NCA biomarker identified when all databases were combined. Panel **A** shows the genes and network identified. The connections correspond to data from the PPI database used. The most connected genes were PRKACA, ESR1, LRP1, SMAD2 and JUN. Panel **B** shows the risk group prediction (splitting the prognostic index by the median) of the multi-NCA biomarker across cancer datasets. Panel **C** depict the color-coded differential expression of genes between risk groups. Darker red indicates more significant differences. The scales were estimated in -Log_10_ of the *t* test p value. Only p values <0.01 are highlighted. Darker purple indicates more significant hazard ratio associations within the Cox model. The scales were expressed in -Log_10_ of the Z p value. Only p values <0.05 are highlighted. Panel **D** shows, in the top, the curated biological terms and pathways associated with the genes composing the biomarker. The associations of genes with specific cancers based on the literature are shown at the bottom.

The identified network biomarker was composed of 41 genes highly connected by ESR1, PRKACA, LRP1, JUN and SMAD2 (Fig. 3A). This gene signature was able to discriminate between low- and high-risk groups efficiently in the 11 cancer datasets (Fig. 3B and Table 3) through the statistical association of specific genes (Fig. 3C). The log-rank test and the Cox model fitting were highly significant across cancer types (Table 3). The average C-index value across cancer types was 0.81 ranging from 0.65 to 1. Eight of these 11 predictions were significant according to a randomization analysis (Fig. 2B and Supplementary Table 3). The highest C-index predictions were observed for BLCA and COADREAD, whereas the lowest C-index predictions were observed for GBM and OV.

In a comparison of the predicted low- and high-risk groups (splitting the prognostic index by the median), we observed several genes differentially expressed across cancer types, except in BLCA (Fig. 3C and Table 3). Apart from LMO4 and DDX5, the other 39 genes were differentially expressed between risk groups in two or more cancer types. LMO4 was not differentially expressed in any cancer but was significantly associated with GBM, LUAD, and LUSC according to the Cox model. DDX5 was highly differentially expressed in LUAD and associated with three cancer types according to the Cox model. Similarly, 36 genes were associated with the Cox model for two cancer types or more. Surprisingly, ESR1 was not associated with Cox models but was differentially expressed in two cancer types and served as a hub for connecting 10 genes.

An overrepresentation analysis of the 41 genes using MSigDB^57^ and DAVID^58^ revealed important biological associations across pathways, transcriptional control, gene ontologies, and other biological terms (Fig. 3D). Some of these pathways are well known to be associated with cancer, such as the MAPK^59^, LKB1^60^, ERα^61^, and NGF^62^ pathways. Some genes were highly associated with transcription factors such as SP1^63^, gene ontologies such as *signaling*, and other biological terms such as *immune system*, *copy number gains in cancer*, and *MIR-18 targets*. In addition, at least 36 of the 41 genes have been associated in the literature with one or more cancer types (Fig. 3D).

These findings support the utility of determining which genes predicted specific cancer types and suggest that the signature we generated is robust across cancer subtypes.

### Comparison of the multi-cancer and cancer type-specific biomarkers

It has been proposed that molecular processes may be similar across cancer types^14^,^15^,^64^. Consequently, a biomarker of clinical outcomes in a specific cancer type may be a good biomarker in a different cancer type. Therefore, we compared our 12 biomarkers to identify similarities. In terms of gene content (Supplementary Table 2), the multi-cancer (multi-NCA) biomarker was not particularly similar to the cancer type-specific biomarkers (Fig. 2A). Indeed, the biomarker most similar to others was OV, which contained 17 genes (29 occurrences) that overlapped with other biomarkers out of the 418 unique genes. This similarity was considerably higher than that of the multi-NCA, which had 9 genes (17 occurrences) overlapping and that of the most specific biomarker, GBM, which had only 4 genes (5 occurrences) in common with the other biomarkers.

A comparison of the average C-index values across biomarkers and cancer types showed that the multi-NCA biomarker was, overall, the best (average C-index = 0.81) but it was closely followed by OV and BRCA (average C-index of 0.80 and 0.79 respectively; Fig. 2). The C-index of the multi-NCA biomarker was almost always better than those of the cancer type-specific biomarkers (Fig. 2D). Nevertheless, in each cancer type, the C-index was higher using the cancer type-specific biomarker than using the multi-NCA biomarker (by 0.047 on average). Despite this, an analysis of 10,000 random biomarkers showed that 8 of 11 C-index predictions of the multi-NCA biomarker were significant (Fig. 2B and Supplementary Table 3) whereas the C-indexes in most cancer type-specific biomarkers were significant only in one or two cancer types (OV in three and marginally in two more). In terms of prediction power per cancer type, the BLCA and COADREAD average C-index values were, by far, the highest (both 0.98). In contrast, the C-indexes for GBM and OV were the lowest (0.62 and 0.65 respectively).

### Comparisons with clinical features

Although biomarkers can be a useful clinical tool to predict outcomes, some of the generated biomarkers may not actually be useful in clinical practice if the gene signature does not add predictive power beyond that of the usual clinical features^30^. To assess this, we determined the C-index of the multi-NCA biomarker and the available clinical features per cancer type. The Supplementary Figure 1 shows that the multi-NCA biomarker adds between 0.04 and 0.30 of prediction power over clinical features alone. In contrast, the clinical features add only between 0 and 0.075 over the biomarker alone. Overall, in most cancer types (except KIRC) the biomarker makes better predictions than clinical features alone. These results suggest that the multi-NCA signature adds a considerable level of predictive power to clinical features.

We also determined whether the multi-NCA signature was sensitive to stratifications using cancer features. For this, we used the widely used cancer staging system for each cancer type to compare the performance of C-indexes across cancer stages. As shown in Supplementary Figure 2, C-index values varied somewhat across stages, perhaps influenced by the number of TCGA samples available per stage. Of note, in BRCA stage IIIA, in KIRC stage III and IV, OV stage IIIB, and UCEC stage IIIC, the C-indexes were lower than 0.05 relative to the overall C-indexes for corresponding cancer types (in these cases the estimation considered more than 20 samples). Nevertheless, the C-index value is still acceptable for most stages. This stratification provides an estimation of the response of markers across a wide spectrum of subtypes.

### External comparison and validation of biomarkers

For external validation of the multi-NCA biomarker, we compared the C-index with other 5 multi-cancer biomarkers proposed by other authors^32^,^65^,^66^ representing signatures of chromosome instability (CIN70)^32^, multiple cancer-related pathways (poised gene cassette, PGC)^65^, mesenchymal transition (MES)^66^, mitotic chromosomal instability (CIN)^66^, and lymphocyte infiltration (LYM)^66^. The 41 genes in the multi-NCA biomarker did not overlap with any of the genes in CIN70, PGC, MES, CIN, and LYM (Supplementary Table 2). The average C-index for the LYM biomarker was 0.796, just below that of our multi-NCA biomarker, which was 0.809 (Supplementary Table 3). The C-index for LYM was nevertheless significant in only 3 TCGA datasets compared with 8 datasets for the multi-NCA biomarker, suggesting that our multi-cancer biomarker is superior to the LYM biomarker.

**Figure 4:**
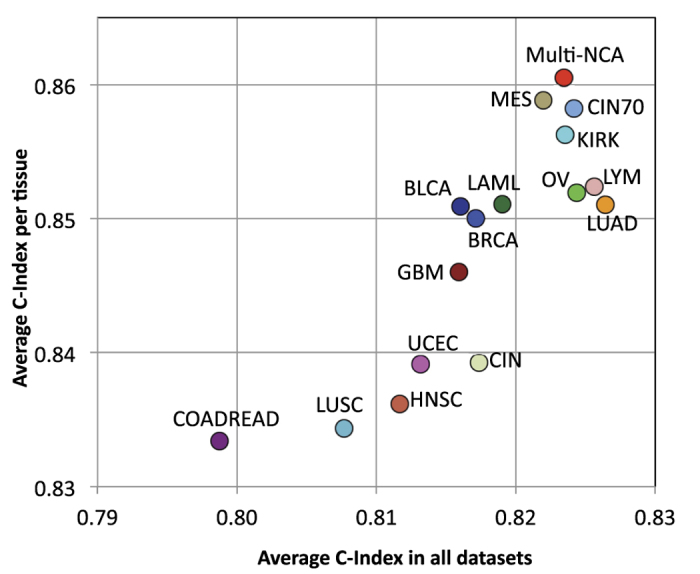
Evaluation of all biomarkers in SurvExpress using C-index. PGC biomarker derived from other authors is not shown (0.74 for all datasets and 0.81 for per tissue) to emphasize biomarkers with higher C-index values.

To evaluate the prediction accuracy of the biomarkers in cancer data other than TCGA, we used SurvExpress^56^ to analyze the multi-NCA and the cancer type-specific biomarkers we generated, and the multi-cancer biomarkers generated by other-authors. We used 122 cancer datasets containing 19,105 samples spanning about 20 types of tissues (Supplementary Table 4). These datasets covered cancer types not used to develop the NCA-based biomarkers such as cancer of the bone, esophagus, eye, liver, prostate, pancreas, and skin, as well as medulloblastomas and astrocytomas, and others. We performed two analyses, the first averaging all 122 datasets, and the second normalizing the average per tissue. The second analysis was more important because some tissues have been more studied than others such as lung, ovary, breast, brain, and colon. In addition, some cohorts are reported in various datasets. The results showed that our multi-NCA biomarker was one of the top biomarkers evaluated; it was the most accurate in the per-tissue analysis and close to the most accurate in all datasets (Fig. 4 and Supplementary Table 4). Compared with other multi-cancer biomarkers, our multi-NCA signature was more accurate in the per tissue analysis than the CIN, CIN70, PGC, LYM, and MES signatures. Among these, the MES was the best in the per-tissue analysis while LYM was first considering all datasets.

### Comparison of multi-cancer module evaluation functions

The results reported here represented by the multi-NCA biomarker were obtained using GBM as the reference cancer type minus the range of all other cancer types examined. We also explored the performance of the network-based marker generation using other functions and other cancer types as reference. We first tested the obvious average function, followed by the average minus the range. As demonstrated in Supplementary Figure 4, using only the average function generated the poorest performance, which was improved by subtracting the range but still lower than using GBM as the reference minus the range. Then, we tested the other three cancer types used as references: LUAD, OV, and BRCA. Interestingly, using LUAD as the reference generated a lower performance than that of GBM in all cancer types, whereas using OV generated almost the same overall performance as GBM. Surprisingly, using BRCA as the reference resulted in a better performance than that of GBM in 7 cancer types (only LUAD showed a decrease; the overall increase in performance was 0.025).

## Discussion

We used NCA, a network-based algorithm, to identify biomarkers highly predictive of survival outcomes in cancer. We first identified biomarkers for specific cancers and then identified a multi-cancer biomarker for 12 cancer types. Interestingly, the gene content varied greatly across biomarkers but the performance was similar when evaluated in each cancer type (Fig. 2D). These results suggest that C-index values are more dependent on cancer type than on gene content of the biomarker. Consequently, survival outcomes may be more difficult to predict in some cancer types than in others. For instance, survival was easier to predict in BLCA and COADREAD than in OV and GBM. This is also supported by the fact that C-index values close to 1 for BLCA and COADREAD were not significant since random markers also showed high C-index values while C-indexes of 0.66 for OV and 0.65 in GBM were highly significant compared with random markers.

The OV-NCA biomarker was the second most accurate biomarker across cancer types (Fig. 2C) even though it was developed using the ovarian serous cystadenocarcinoma dataset only. A comparison of the OV biomarker (Supplementary Figure 3) with the multi-NCA biomarker (Fig. 3) showed that, surprisingly, the ovarian biomarker had more connections than the multi-NCA biomarker. However, the number of differentially expressed genes, the Cox model statistics, and the biological terms associated with the signature were more appropriate in the multi-NCA biomarker than in the OV biomarker. The multi-NCA was able to significantly predict survival outcomes in 5 more cancer types than the OV biomarker (Fig. 2B), and it was more accurate in the per-tissue analysis (Fig. 4) than the OV biomarker. These findings indicate that the multi-NCA biomarker was more suitable for multi-cancer predictions than the OV biomarker. Nevertheless, it would be interesting to explore why the OV biomarker was highly predictive of outcomes across cancers. Although ovarian cancer was hard to predict, glioblastoma was even harder but the GBM biomarker was less accurate than the OV biomarker (Figs 2C and 4), so it cannot be easily linked to prediction difficultness. Ovarian serous cystadenocarcinoma can be divided into various subtypes defined by immunoreactive, mesenchymal, proliferative, and differentiated characteristics^6^. These characteristics represent universal tumorigenic processes and are observed in other types of cancer as well^6^. This heterogeneity is reflected in the relatively high number of individuals (578) included in the TCGA ovarian dataset^16^, although a similar number of samples was included in glioblastoma and invasive breast carcinoma (Table 1). In addition, the five genes (JUN, PRKACA, SMAD2, ESR1, and BCL3) shared by the OV and multi-NCA biomarkers form a small network module and are recognized as cancer-related genes. Further analysis is needed to explore the reasons for the apparently high inter-cancer accuracy of the OV biomarker.

None of the C-index values in BLCA or in COADREAD were significant in the random model test even though the C-index values reached 1 because 46% and 12% of the random models respectively were equally predictive. Moreover, in BLCA, none of the genes were differentially expressed between risk groups. Although the low number of samples could influence these results (only 54 samples in BLCA and 151 in COADREAD), confirmed results in larger cohorts would imply that many predictive signatures may exist. In our study, the multi-NCA did not depend on the number of samples per cancer type but in the NLLRT of each cancer type. Thus, the selection of the best signature was imposed by other cancer types rather than by BLCA and COADREAD. This may explain why none of the genes was significant in BLCA. Nevertheless, these findings do not necessarily indicate that these genes in BLCA are not important. For instance, high expression of CALR has been associated with high risk in bladder cancer^67^. HRAS gains have been found in bladder cancer cell lines and have been related to urothelial tumorigenesis^68^. ITGA4 is part of a methylation gene set used for the detection of bladder cancer^69^. In COADREAD, AKT isoforms (including AKT1) are associated with high expression of CD133 and CD44 (cancer stem cell markers) and radiation resistance in colon cancer cells^70^. High expression of DDX5 (previously known as p68) is related to the transition from polyp to adenoma and then to adenocarcinoma^71^. High levels of DUT protein expression are predictive for tumor resistance to chemotherapy in colorectal cancer^72^. Finally, up-regulation of JUN is related to the invasiveness of colorectal cancer cells. These findings clearly indicate that the biomarker genes are biologically related to BLCA and COADREAD.

The performance comparisons of the multi-NCA with clinical features suggest that the multi-NCA signature adds predictive power to clinical features. Nevertheless, these comparisons also showed that the predictive power of the multi-NCA biomarker might vary across cancer stages. This may indicate that the biomarker is somehow influenced by the high representation of specific cancer subtypes in the TCGA studies. For example, the results in BRCA were highly influenced by stage II samples, which accounted for more than 50% of total samples, whereas stage IV samples represented only 3% of samples. Other cancer types showed similar staging biases. Inclusion of more samples (as is happening with the TCGA and the International Cancer Genome Consortium datasets) and prefiltering of data to balance stage representation may be good strategies to improve the identification of multi-cancer biomarkers.

The C-index value of our multi-NCA biomarker was higher than that of other previously reported multi-cancer biomarkers, but not substantially. The C-index values of MES and CIN70 were just below that of our multi-NCA biomarker. Some of the other multi-cancer biomarkers however use more genes for the prediction (Supplementary Table 2). Still, these comparisons highlight the fact that our multi-NCA biomarker is highly competitive among the others reported.

The network-based strategy that we used emphasizes the fact that using biological information coupled with gene selection is a powerful strategy to generate biomarkers; this conclusion is consistent with results from other studies^46^,^47^,^48^,^49^,^50^,^51^. However, the network-based strategy that we used is different from other approaches in various ways (Supplementary Table 5). First, we directly evaluated a Cox model that is capable of identifying combinatorial features more robustly than univariate-oriented approaches^47^, classifiers^46^,^49^,^51^ or components^48^. Second, unlike in other algorithms, we did not prefilter genes to decrease the complexity of the exploration^47^. Third, we used population-dependent selection of the most improved models allowing us to explore more combinations than would be possible using other algorithms^46^,^47^. Finally, to generate a multi-cancer biomarker, we expanded the Cox evaluation to multiple datasets by subtracting the range of all NLLRT values from the NLLRT value of a reference cancer.

We used the HPRD protein-protein interaction network in our approach. In principle, however, the NCA approach can be applied to other biological networks such BioGrid^73^, iRefWeb^74^, STRING^75^, and to genetic regulatory networks such as MotEvo^76^ and the conserved transcription factor binding sites track in UCSC (https://genome.ucsc.edu). The NCA algorithm is not limited to gene expression data or to survival analysis as the response variable. The exploration of diverse biological networks, genomic data, and response variables may lead to the identification of better or alternative multi-cancer biomarkers.

The identification of novel or alternative multi-cancer biomarkers is also valuable because such biomarkers can represent different biological phenomena that may help to elucidate specific cancer features. For example CIN70 was identified from chromosome instability^32^, MES from mesenchymal transition^66^, and LYM from lymphocyte infiltration^66^. Our multi-NCA biomarker represents a protein-network-based biomarker. In this context, our multi-NCA biomarker does not share genes with other multi-cancer markers and shares only 5 genes with the OV biomarker also identified here.

We tested diverse module evaluation functions in which we varied the reference cancer type. We observed that the biomarkers found, and their performance depended on this evaluation. These results have deep implications: the choice of the module-growth function is critical, the function used can be improved, and the approach can generate alternative markers. It would be interesting to explore other functions combined with more cancer types.

Recent results have explored the correlation between gene expression and genomic changes such as copy number alterations^77^. In this context, the predictive power of the multi-NCA biomarker appeared to be specific for gene expression because mutations and copy number alterations were not highly related (Supplementary Table 6). The search for mutation signatures associated with clinical outcomes is starting^30^. Given the sparseness of the mutational spectrum across cancers, it is difficult to realize that a general mutation signature could be found. It would be exciting to see whether approaches like our proposal are capable of providing interesting solutions.

The identification of multi-cancer biomarkers may lead to proposals of novel diagnostic tools and therapeutic schemes. In this context, using DGIdb^78^ we observed that 22 of the 41 genes of the multi-cancer biomarker were known drug targets (Supplementary Table 7). Thus, our approach may also shed light on which targets can be assayed in future experiments.

## Additional Information

**How to cite this article**: Martinez-Ledesma, E. *et al.* Identification of a multi-cancer gene expression biomarker for cancer clinical outcomes using a network-based algorithm. *Sci. Rep.* **5**, 11966; doi: 10.1038/srep11966 (2015).

## Supplementary Material

Supplementary Information

## Figures and Tables

**Table 1:** Cancer datasets used for our study.

**ID**	**Type**	**Samples/ Censored**	**Platform**
BLCA	Bladder Urothelial Carcinoma	54/35	RNA-Seq
BRCA	Breast Invasive Carcinoma	502/437	Agilent
COADREAD*	Colon and Rectum Adenocarcinoma	151/134	Agilent
GBM	Glioblastoma	538/116	Affymetrix
HNSC	Head and Neck Squamous Cell	283/164	RNA-Seq
KIRC	Kidney Renal Clear Cell	468/313	RNA-Seq
LAML	Acute Myeloid Leukemia	168/60	RNA-Seq
LUAD	Lung Adenocarcinoma	255/175	RNA-Seq
LUSC	Lung Squamous Cell	205/120	RNA-Seq
OV	Ovarian Serous Cystadenocarcinoma	578/276	Affymetrix
UCEC	Uterine Corpus Endometrial Carcinoma	333/305	RNA-Seq
MULTI	All cancers above	3535/2135	

^*^Colon and rectal adenocarcinoma datasets (COADREAD) were fused as in the TCGA pan-cancer analyses.

**Table 2:** Networks modules obtained for each cancer type using the NCA algorithm.

**Type**	**Modules**	**Module Size**	**Network-based Biomarker**	
			**Size**	Top Genes*
BLCA	10,303	10	41	**SMAD2**, RUNX2, ABTB1, ST5, CEBPB, SETDB1, CEBPG
BRCA	485	9	42	JAK2, NFKBIA, **TBP**, RXRA, VAV1, HES5, NFKBIB
COADREAD	252	13	36	**EEF1A1**, FOXG1, GADD45G, MAPK9, MYOC, **SMAD2**
GBM	2,142	9	42	EFEMP2, MAPK3, TP53, TOP1, CCDC6, SREBF1, GJA1
HNSC	661	9	41	DUSP16, KRT8, RAF1, MED1, PPARG, YWHAB, FABP1
KIRC	2,841	4	41	AR, HGS, RUNX1, **BCL3**, **BRCA1**, STAT2, ITGA8
LAML	584	8	42	GUCY2C, PTPRA, SRC, STAT5B, WAS, KCNQ5, CALM1
LUAD	808	9	42	DOK1, FUT4, INSR, ITGB2, SHC1, PTPRC, KHDRBS1
LUSC	84	14	37	**BRCA1**, ETS2, HIF1A, **JUN**, LMO4, PIAS3, RBBP7
OV	421	9	42	**TBP**, LCK, ESR2, RB1, **JUN**, **EEF1A1**, **BCL3**
UCEC	1,570	10	41	CREBBP, GTF2B, CSNK2A1, CTNNB1, HOXD4, HIPK1, PTEN
MULTI	2	44	41	**ESR1, PRKACA, LRP1, **JUN**, **SMAD2**, SNAP25, ITNS1

^*^The complete lists of genes and samples used are shown in Supplementary Table 1.

^**^Highest connected genes. Genes in boldface type are repeated more than once in this list.

**Table 3:** Cox model results showing how well the multi-NCA cancer biomarker fit across datasets.

**Cancer Type**	**C-index**	**Log Rank Test**	**Cox p-Value**	**Significant Genes**	**Differential Expressed Genes**
BLCA	1.00	6.3	3.3	0	0
BRCA	0.80	9.4	7.8	11	17
COADREAD	1.00	4.7	2.5	7	8
GBM	0.65	5.8	5.7	8	19
HNSC	0.75	7.7	7.0	9	15
KIRC	0.74	8.7	8.1	5	21
LAML	0.77	11	9.6	9	10
LUAD	0.81	5.1	4.7	13	16
LUSC	0.77	8.5	7.4	13	9
OV	0.66	9.2	5.8	11	18
UCEC	0.92	5.7	3.5	19	13
