# Supplementary material for: Identification of a multi-cancer gene expression biomarker for cancer clinical outcomes using a network-based algorithm
Source: Sci Rep. 2015 Jul 23;5:11966. doi: 10.1038/srep11966 (PMC5378879; doi:10.1038/srep11966)
Supplement: Supplementary Information [file srep11966-s1.pdf]

SUPPLEMENTARY INFORMATION

Identification of a multi-cancer gene expression biomarker for cancer clinical outcomes using a network-based algorithm

Emmanuel Martinez-Ledesma[1,2], Roeland G.W. Verhaak[2,3], Victor Treviño[1]
emmanuelmtz@gmail.com, rverhaak@mdanderson.org, vtrevino@itesm.mx
[1] Grupo de Enfoque e Investigación en Bioinformática, Departamento de Investigación e Innovación, Escuela Nacional de Medicina, Tecnológico de Monterrey, Monterrey, Nuevo León 64849, México.
[2] Department of Genomic Medicine, The University of Texas MD Anderson Cancer Center, Houston, Texas 77030, USA.
[3] Department of Bioinformatics and Computational Biology,
The University of Texas MD Anderson Cancer Center, Houston, Texas 77030, USA.

A

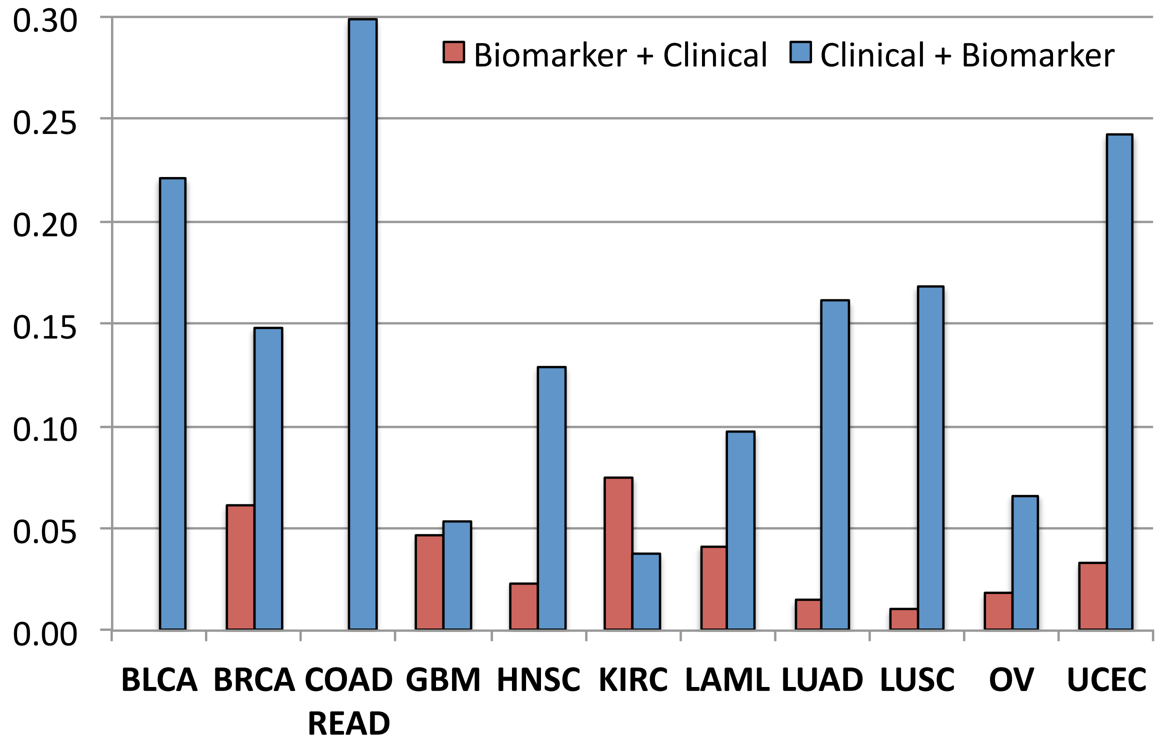

B

Dataset	Multi-NCA	Clinical	Both	Clinical Features
**BLCA**	1.000	0.779	1.000	Age + AJCC_Stage + AJCC_Stage_Code
**BRCA**	0.805	0.718	0.866	Age + PR + ER + AJCC
**COADREAD**	1.000	0.702	1.000	Age + Colon_Polyps + Pathology + Stage
**GBM**	0.648	0.641	0.694	Age
**HNSC**	0.755	0.649	0.778	Age + Grade + Pathology + Stage
**KIRC**	0.738	0.775	0.813	Age + Grade + Pathology + Stage
**LAML**	0.777	0.721	0.818	Age + Cyto_Risk + Morpho_Code
**LUAD**	0.814	0.667	0.828	Age + Stage + Pathological_Stage
**LUSC**	0.773	0.615	0.783	Age + Stage + Pathological_Stage
**OV**	0.664	0.616	0.682	Age + Stage
**UCEC**	0.926	0.717	0.959	Age + Grade + Figo_Stage

Supplementary Figure 1. Improvement in C-index using clinical features. Panel A shows the improvement in C-index of the multi-NCA biomarker when clinical features are added to the model (red) and the improvement in C-index of the clinical features when the biomarker is added to the model (blue). Panel B shows the C-index evaluations using the biomarker, the clinical features, and both. The clinical features used for each cancer type are also included.

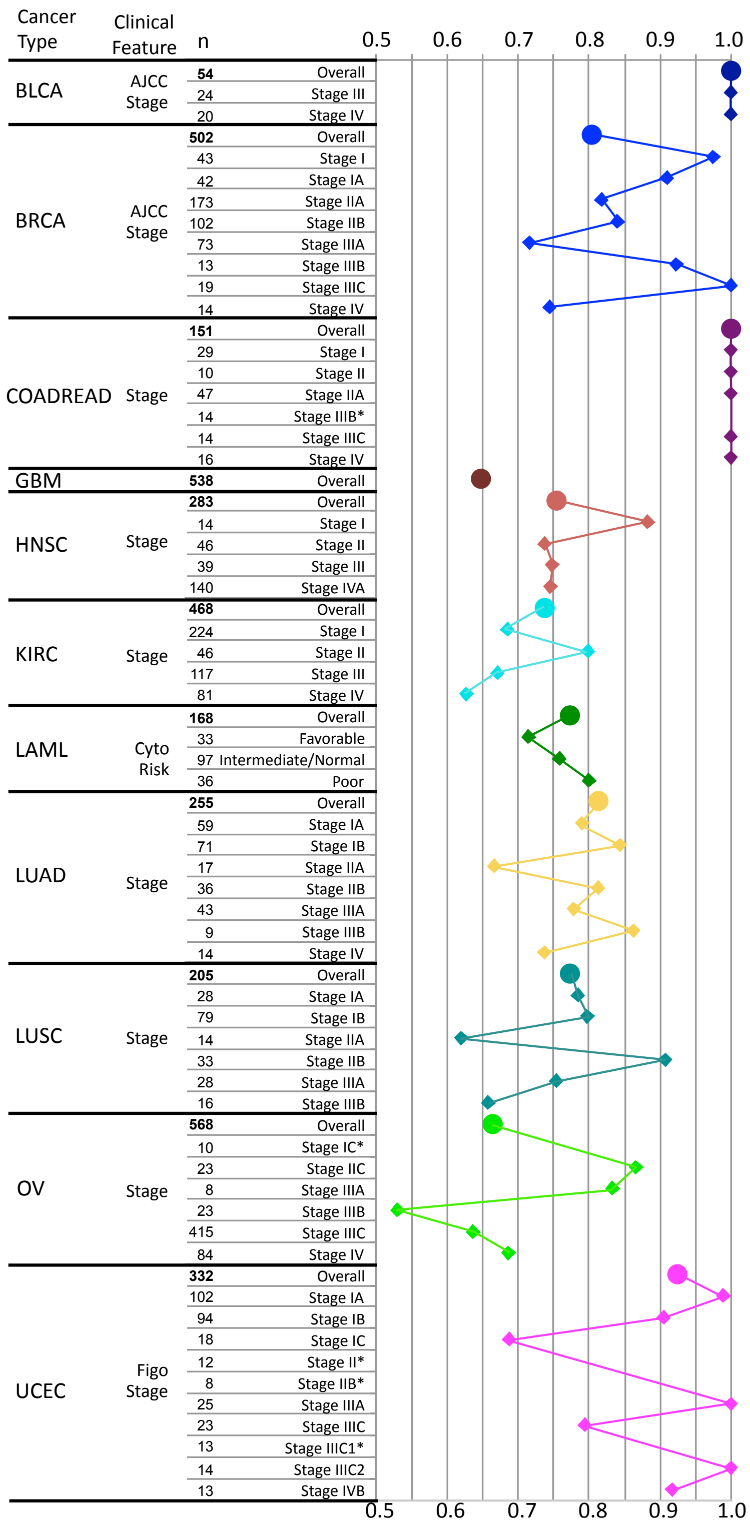

Supplementary Figure 2. C-index performance of the multi-NCA biomarker across cancer stages for each cancer type. Bigger circles denote the overall C-index. * Indicates stages that could not be estimated in SurvExpress because all samples were censored or the number of samples was too low.

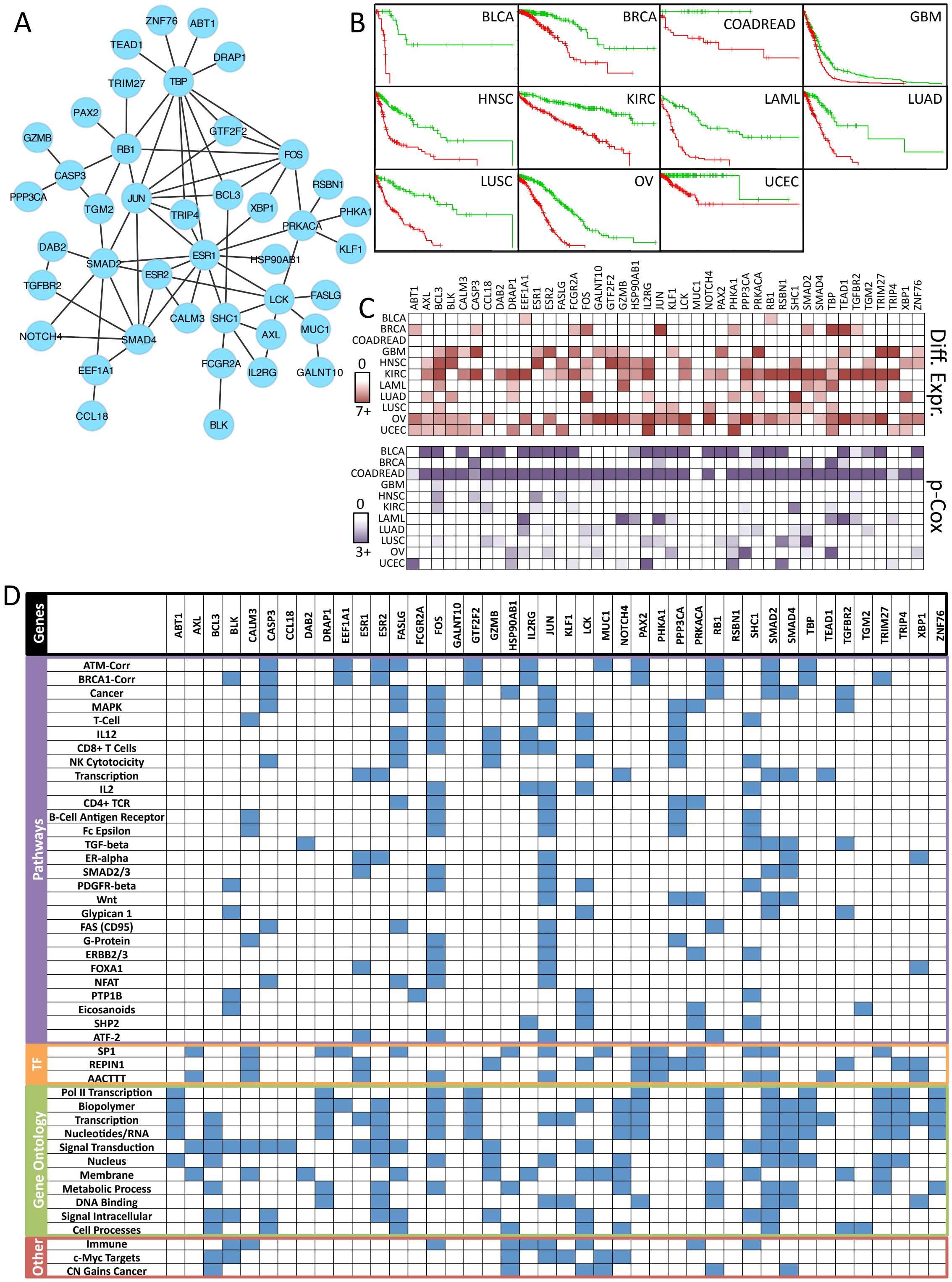

Supplementary Figure 3. Ovarian biomarker. Panel A shows the genes and network identified. The connections correspond to data from PPI database used. Panel B shows the Kaplan-Meier curves of the risk group prediction across cancer

datasets. Panel C depict the color-coded differential expression of genes between risk groups. Darker red indicates more significant differences. The scales were estimated in $-\text{Log}_{10}$ of the *t* test p value. Only p values < 0.01 are highlighted. Panel C also displays the color-coded associations among genes within the Cox model. Darker purple indicates more significant hazard ratio associations. The scale is expressed in $-\text{Log}_{10}$ of the Z p value. Only p-values < 0.05 are highlighted. Panel D shows, in the top, the curated biological terms and pathways associated with the genes composing the biomarker.

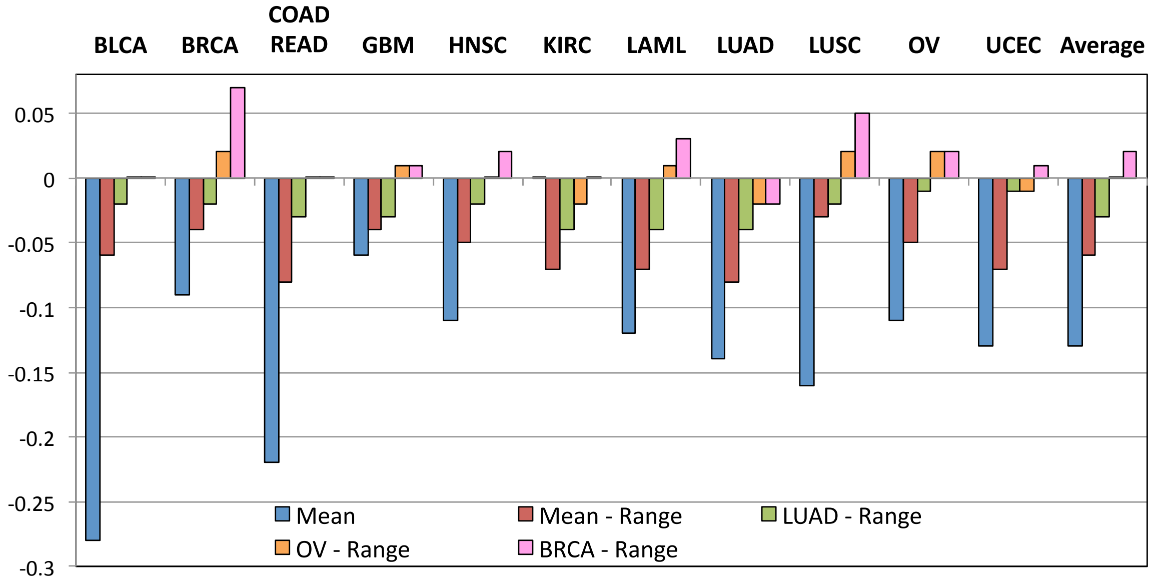

Supplementary Figure 4. C-index improvement of generated biomarkers using alternative cancer reference or function. The figure shows the improvement in C-index (vertical axis) across cancer types (horizontal axis) when other references or functions are used in the network growing function. Zero in C-index improvement denotes an equivalent result to the GBM-range(NLLRT) used in our study. Mean represents no range subtraction. Mean – Range indicates that the function depends on the average minus the difference between the maximum prediction and minimum prediction across cancer types. *OV - range, BRCA - range,* and *LUAD - range* represent the Ovarian Cancer NLLRT, Breast Cancer NLLRT, and Lung Adenocarcinoma NLLRT respectively minus the NLLRT range of remaining cancer types. The average bars group at the right represents the overall difference. The change in prediction is a consequence of the resulting markers.

Supplementary Table 1. Genes and samples used in our study.

The complete list of genes and samples used in the study can be obtained from our web site http://bioinformatica.mty.itesm.mx/multicancer-biomarker.

Supplementary Table 2. Genes included in all signatures used.

BLCA	BRCA	COADREAD	GBM	HNSC	KIRC	LAML	LUAD	LUSC	OV	UCEC	MULTI	LYM	CIN	MES	CIN70	PGC
ABTB1	AOC3	ACP1	A2M	APP	ANK3	ABL1	ANP32A	ARL4D	ABT1	ATF1	ACVR1B	ABI3*	ASPM	ADAM12	ACTL6A	AASDHPPT
ATF4	BCR	BCL2	ATF7IP	BRCA1	AR	ANKRD11	CBL	BRCA1	AXL	CDH6	AKT2	AIF1	AURKA	ADAMTS12	ASF1B	ACTG1
BRCA1	CAPN2	CAP1	ATXN1	CASP2	BCL3	BCR	CD226	C22orf46	*BCL3*	CDH9	BCL3	APBB1IP	AURKB	ADAMTS2	ATAD2	C1orf37*
BTBD2	CASP3	CDK4	C2orf3	CAV1	BRCA1	CALM1	CXCR4	C2orf88	BLK	CDK5RAP3	C2	ARHGAP9*	BUB1	AEBP1	AURKB	CPSF6
C7orf64	CSNK2A1	CRADD	CASP3	CD19	CHEK2	CNTNAP1	CYTH2	CTBP1	CALM3	CDKN1A	C3	BIN2	BUB1B	ANGPTL2	BRRN1*	DYRK1A
CALM1	DRAP1	CTNNB1	CCDC6	CFLAR	CNTNAP1	CRKL	DHX9	DLL4	CASP3	CNOT3	CALR	BTK	CCNA2	ANTXR1	C20orf24	ETF1
CAMK2A	EDF1	DUSP19	CD40LG	CNKSR1	COL7A1	CSK	DOK1	ETS2	CCL18	CREBBP	CCNH	C1QA	CCNB2	ASPN	CCNB1	FLJ10276*
CBFB	EPHA3	DUSP4	CDH5	CRKL	CREBBP	CSNK2A2	EZR	GRIPAP1	DAB2	CSNK2A1	CFTR	C1QB	CDC20	BGN	CCNB2	FLJ20321*
CD44	EZH2	EEF1A1	COL4A2	DNMT1	CSNK2A1	DGKZ	FLT1	HDAC3	DRAP1	CTNNB1	CIITA	C3AR1	CDC6	BNC2	CCT5	FLJ20323*
CD74	FOS	ESR2	DHFR	DUSP16	EP300	EP300	FUT4	HIF1A	EEF1A1	ESR1	CTGF	CCR5	CDCA5*	CDH11	CDC2*	FLJ20628*
CEBPB	GTF2A1	FOXG1	DNAJC1	ELK1	FN1	ERBB4	GAB2	ING1	*ESR1*	FAF1	DDX5	CD2	CDCA8	COL10A1	CDC20	FUS
CEBPG	HES5	GADD45G	DUSP1	ESR1	FOXM1	GAB2	GHR	JUN	ESR2	FGF2	DUT	CD37	CENPA	COL11A1	CDC45L*	GMFB
CHEK1	HIPK2	GATAD2B	EFEMP2	FABP1	HBP1	GCSH	GNB2L1	LMO4	FASLG	FOS	ESR1	CD3E	CENPF	COL1A1	CDC6	GTF3C2
CSF1	IGF1R	ITGA7	EIF3G	FAM82A2	HDAC1	GUCY2C	GRB2	MED1	FCGR2A	FOXF2	HMGN1	CD4	CEP55	COL1A2	CDCA3	HNRPR*
DCP1B	JAK2	MAPK1	FAM46A	HSPA4	HGS	HSP90AA1	HNRNPC	MED15	FOS	GATA1	HRAS	CD48	CKAP2L*	COL3A1	CDCA8	HSA9761*
EGFR	LEPR	MAPK3	FTL	JAK1	HMGA1	KCNQ5	ICAM3	MITF	GALNT10	GRIP1	ITGA4	CD53	DEPDC1	COL5A1	CEP55	ITSN1
EIF3D	MCM5	MAPK9	GJA1	KIF1A	IFI16	KIT	IL2RG	NCOA2	GTF2F2	GTF2A1	ITSN1	CYBB	DLGAP5*	COL5A2	ch-TOG*	K-ALPHA-1*
EIF3L	MYOD1	MYOC	HSPA1A	KRT8	ITGA8	LYN	INSR	NOTCH4	GZMB	GTF2B	JUN	CYTH4*	EXO1	COL6A1	CKS2	KIAA0247
EIF4EBP1	NFKBIA	PPP2R1A	HSPA5	MAP2K7	ITGB1	LYST	IRS1	NR3C1	HSP90AB1	HIPK1	KCNB1	DOK2*	FOXM1	COL6A2	CMAS	KS*
ESR2	NFKBIB	PRKCE	HSPB8	MAPK1	KAT2A	MAPK1	ITGB2	OS9	IL2RG	HOXD4	KCNJ12	EVI2B	GTSE1	COL6A3	CNAP1*	MAP3K11
HDAC1	NKIRAS1	PSMD8	IER3	MAPK8	KAT5	MAPKAPK5	ITGB7	PIAS3	*JUN*	IRF3	LGALS8	FCER1G	HJURP*	COL8A1	CTPS	MARCH3
HHEX	NKIRAS2	PTN	KIAA0408	MAPK8IP3	NCOA4	MATK	JAK2	POU1F1	KLF1	KLF5	LMO4	FERMT3*	KIF11	CRISPLD2	DHCR7	MATR3
INSR	PKN2	RAB27A	LAMA4	MAPK9	NFKB1	MED28	KHDRBS1	PRKAR1A	LCK	LIG1	LRP1	FGL2	KIF14	CTSK	DKC1	MFAP3*
JUN	PPP2R5A	RARA	MAPK3	MAPKAP1	PDGFRB	MME	KRT27	PSMD11	MUC1	MED25	LRPAP1	GIMAP4	KIF15	EMILIN1	ECT2	MRCL3*
KPNB1	RAC1	RASL12	MPHOSPH6	MAPKSP1	PIAS1	MST1R	LCK	RARB	NOTCH4	NOS2	MAP2	GIMAP5	KIF18B*	FAP	ELAVL1	MTCH2
MAPK1	REL	RXRA	NCL	MARK4	PIK3R1	NMI	LTK	RBBP7	PAX2	PAK1	MARK4	GPR65	KIF20A	FBN1	ESPL1	MUS81
MMP7	RELA	SMAD2	NUP85	MDM2	PLK1	NPHS1	LYN	RBP1	PHKA1	PDPK1	MLL2	HAVCR2*	KIF23	FNDC1*	EZH2	NACA
MRPL44	RPS6KA1	SMAD3	PIAS4	MED1	PTPRB	PELP1	MED28	RFC1	PPP3CA	POLR1B	MMP15	IL10RA	KIF2C	GLT8D2	FEN1	NFRKB
NIPSNAP3A	RXRA	ST13	PPP2CA	MFAP1	RAN	PIK3R1	NGFR	SAP30	*PRKACA*	PPP1R1B	MTCH2	IL21R	KIF4A	ISLR	FLJ10036*	OXSR1
PIAS3	SNAPC2	STMN2	RAD54L2	NR2C2	RANGAP1	PKD1	NPM1	SMAD2	RB1	PTEN	NEDD9	ITGAL	KIFC1	ITGA11*	FOXM1	P53CSV*
PSMD11	SPIB	TAL2	RCHY1	PPARG	RORA	PPEF2	PDGFRB	SMURF2	RSBN1	PTPRZ1	PDC	ITGB2	MCM10	LRRC15	GPI	PRO2730*
RUNX2	STAM2	TEX11	RIBC2	PRLR	RUNX1	PTPRA	PLSCR1	ST13	SHC1	PYGO1	PDIA3	LAIR1	MELK	LUM	H2AFX	RBBP6
SETDB1	STAT5A	TSN	SCAMP1	PXN	SHC1	RAF1	PPFIA1	TCF4	*SMAD2*	SLC18A2	PRKACA	LAPTM5	MYBL2	MMP2	H2AFZ	RFP2*
SMAD2	TAF7L	TSNAX	SREBF1	RAF1	SRC	SRC	PRKCB	TPM2	SMAD4	SLC9A3R1	PTPN7	LCP2	NCAPG*	NID2	HDGF	RHOG
SMAD4	TBK1	TTR	TCF3	RAP1A	STAT2	STAT5B	PTPN6	VHL	TBP	SOX1	RPS6KA1	LILRB1	NCAPH	OLFML2B	KIAA0286*	RPL7A
SNRNP70	TBP	ZCCHC10	TOP1	RASSF1	STAT3	STAT6	PTPRC	VHLL	TEAD1	SRC	RRAD	MPEG1*	NDC80	PDGFRB	KIF20A	RPS2
SOCS1	TP53		TOP2B	RB1	TEK	TNFRSF1A	RTN4R	ZNF675	TGFBR2	SS18L1	SLC9A6	MS4A6A	NEK2	POSTN	KIF4A	RY1*
ST5	TUBB		TP53	SRRM2	TELO2	TRAF1	SH2B3		TGM2	TAF4	SMAD2	MYO1F	NUSAP1	PRRX1	LSM4	SEC24B
TNFSF11	TYK2		UBE2A	TUBB	TRAF6	USHBP1	SH2D1A		TRIM27	TAF9	SNAP25	NCKAP1L	ORC1L*	SFRP2*	MAD2L1	SEPT2
TUBA1A	VAV1		UBE2I	YWHAB	VPS37D	VCL	SHC1		TRIP4	THRA	TH	PLEK	PLK1	SPARC	MCM10	SKIIP*
VCAN	XRCC4		VIM	YWHAG	ZBTB16	WAS	STAT5A		XBP1	TRAT1	TOP1	PTPRC	PRC1	THBS2	MCM2	SLC35D1
	ZYX		WWOX			YWHAZ	TRA2B		ZNF76			SASH3*	RACGAP1	THY1	MCM7	SMNDC1
												SELPLG	RRM2	TIMP2	MELK	STAU*
												SLA	SGOL1*	VCAN	MGC13096*	TSC1
												SLAMF8	TOP2A		MSH6	UBB
												SNX20*	TPX2		MTB*	UBC
												SPI1	TROAP		MTCH2	VPS26*
												SPN	TTK		NDUFAB1	ZNF45
												TYROBP	UBE2C		NEK2	
												WAS			NUP205	
															NXT1	
															OIP5	
															PCNA	
															PRC1	
															PTTG1	
															RAD21	
															RAD51AP1	
															RFC4	
															RNASEH2A	
															RRM1	
															RRM2	
															SFRS2*	
															STK6*	
															TGIF2	
															TOP2A	
															TOPK*	
															TPX2	
															TRIP13	
															TTK	
															UBE2C	
															UNG	
															ZWINT	

* Not found in the merged TCGA dataset

Supplementary Table 3. C-index and p-values of all signatures.

C-index

	DATASETS												
**Biomarker**	**BLCA**	**BRCA**	**COADREAD**	**GBM**	**HNSC**	**KIRC**	**LAML**	**LUAD**	**LUSC**	**OV**	**UCEC**	**Average**	**Rank**
**BLCA**	1.000	0.709	0.992	0.606	0.671	0.755	0.775	0.763	0.724	0.636	0.872	0.773	11
**BRCA**	0.975	0.900	0.914	0.614	0.747	0.748	0.753	0.791	0.732	0.652	0.924	0.795	4
**COADREAD**	0.992	0.761	1.000	0.592	0.688	0.716	0.734	0.731	0.684	0.656	0.879	0.767	15
**GBM**	1.000	0.741	0.973	0.709	0.738	0.734	0.762	0.772	0.736	0.622	0.833	0.784	8
**HNSC**	0.769	0.707	0.949	0.596	0.836	0.719	0.741	0.780	0.721	0.627	0.862	0.755	17
**KIRC**	0.981	0.804	1.000	0.635	0.722	0.780	0.734	0.791	0.743	0.624	0.875	0.790	5
**LAML**	1.000	0.737	0.852	0.627	0.714	0.744	0.847	0.773	0.713	0.633	0.880	0.774	10
**LUAD**	1.000	0.741	0.900	0.632	0.744	0.735	0.785	0.887	0.697	0.646	0.864	0.785	7
**LUSC**	1.000	0.679	0.958	0.605	0.737	0.721	0.749	0.731	0.870	0.627	0.821	0.773	12
**OV**	1.000	0.797	0.998	0.593	0.751	0.736	0.764	0.756	0.750	0.732	0.946	0.802	2
**UCEC**	1.000	0.729	0.954	0.638	0.655	0.754	0.756	0.786	0.731	0.648	0.996	0.786	6
**MULTI-NCA**	1.000	0.805	1.000	0.648	0.755	0.738	0.777	0.814	0.773	0.664	0.926	**0.809**	**1**
**CIN**	1.000	0.775	0.984	0.626	0.709	0.713	0.776	0.738	0.732	0.633	0.888	0.779	9
**LYM**	0.983	0.779	0.998	0.638	0.721	0.750	0.747	0.729	0.798	0.663	0.953	0.796	3
**MES**	1.000	0.723	0.966	0.626	0.718	0.774	0.719	0.762	0.713	0.615	0.882	0.773	13
**CIN70**	1.000	0.733	0.889	0.619	0.748	0.739	0.763	0.733	0.685	0.629	0.904	0.767	14
**PGC**	1.000	0.741	0.934	0.617	0.691	0.707	0.749	0.743	0.694	0.631	0.882	0.763	16
**Average**	0.982	0.757	0.957	0.625	0.726	0.739	0.761	0.769	0.735	0.643	0.893		
**Rank**	1	6	2	11	9	7	5	4	8	10	3		

(Darker red backgrounds indicate higher statistical significance in both tables)

p values of the C-index from 10,000 Random 41-gene biomarkers

	DATASETS												
**Biomarkers**	**BLCA**	**BRCA**	**COADREAD**	**GBM**	**HNSC**	**KIRC**	**LAML**	**LUAD**	**LUSC**	**OV**	**UCEC**	**P < 0.05**	**Rank**
**BLCA**	0.4628	0.8792	0.3644	0.8756	0.9868	0.0955	0.0367	0.3359	0.3419	0.4764	0.5707	1	6
**BRCA**	0.8349	0.0000	0.8999	0.7067	0.0602	0.2169	0.2554	0.0558	0.2301	0.1277	0.0503	1	6
**COADREAD**	0.6502	0.3573	0.1225	0.9809	0.9240	0.9571	0.6086	0.8149	0.8873	0.0780	0.4711	0	15
**GBM**	0.4628	0.5924	0.6151	0.0000	0.1323	0.6218	0.1320	0.2122	0.1884	0.8083	0.9117	1	6
**HNSC**	0.9829	0.8906	0.7630	0.9615	0.0000	0.9336	0.4741	0.1350	0.3880	0.6795	0.6903	1	6
**KIRC**	0.7924	0.0388	0.1225	0.1781	0.3832	0.0006	0.6039	0.0562	0.1221	0.7528	0.5218	2	4
**LAML**	0.4628	0.6464	0.9886	0.3590	0.5514	0.3339	0.0000	0.2071	0.5228	0.5450	0.4567	1	6
**LUAD**	0.4628	0.5978	0.9358	0.2273	0.0823	0.5869	0.0114	0.0000	0.7526	0.2230	0.6589	2	4
**LUSC**	0.4628	0.9795	0.7154	0.8874	0.1500	0.9109	0.3069	0.8149	0.0000	0.6983	0.9571	1	6
**OV**	0.4628	0.0630	0.2014	0.9750	0.0405	0.5703	0.1077	0.4504	0.0741	0.0000	0.0077	3	2
**UCEC**	0.4628	0.7187	0.7369	0.1227	0.9984	0.1142	0.2010	0.0894	0.2488	0.1943	0.0000	1	6
**MULTI-NCA**	0.4628	0.0371	0.1225	0.0285	0.0267	0.5130	0.0303	0.0038	0.0072	0.0286	0.0445	**8**	**1**
**CIN**	0.4628	0.2083	0.4876	0.3904	0.6461	0.9728	0.0345	0.7305	0.2301	0.5576	0.3510	1	6
**LYM**	0.7767	0.1744	0.2014	0.1212	0.4051	0.1879	0.3538	0.8354	0.0001	0.0326	0.0029	3	2
**MES**	0.4628	0.7753	0.6635	0.3800	0.4730	0.0028	0.8411	0.3527	0.5112	0.9013	0.4378	1	6
**CIN70**	0.4628	0.6863	0.9561	0.5678	0.0531	0.4714	0.1167	0.7901	0.8777	0.6533	0.1851	0	15
**PGC**	0.4628	0.5924	0.8266	0.6397	0.9029	0.9890	0.3108	0.6581	0.7881	0.5972	0.4295	0	15
**P < 0.05**	0	3	0	2	3	2	5	2	3	3	4		

Supplementary Table 4. SurvExpress C-index of all biomarkers used. Panel A, per tissue. Panel B, per dataset.

(A)

Tissue	BLCA	BRCA	COADREAD	GBM	HNSC	KIRC	LAML	LUAD	LUSC	OV	UCEC	MULTI	CIN	LYM	MES	CIN70	PGC
**Bladder**	0.919	0.920	0.896	0.933	0.885	0.929	0.955	0.919	0.896	0.922	0.948	0.910	0.914	0.903	0.906	0.943	0.871
**Bone**	1.000	0.999	1.000	0.998	1.000	0.991	1.000	1.000	0.953	1.000	1.000	1.000	0.999	0.996	1.000	1.000	1.000
**Brain**	0.861	0.859	0.850	0.856	0.852	0.843	0.849	0.850	0.858	0.861	0.859	0.856	0.857	0.850	0.857	0.852	0.819
**Breast**	0.751	0.780	0.768	0.771	0.768	0.764	0.783	0.786	0.763	0.777	0.780	0.810	0.777	0.792	0.778	0.783	0.741
**Colon**	0.815	0.806	0.793	0.784	0.801	0.809	0.798	0.817	0.805	0.814	0.789	0.808	0.826	0.822	0.834	0.812	0.753
**Esophagus**	0.850	0.885	0.844	0.872	0.852	0.891	0.878	0.894	0.867	0.914	0.850	0.901	0.905	0.876	0.888	0.878	0.824
**Head and Neck**	0.847	0.860	0.847	0.885	0.897	0.884	0.879	0.880	0.879	0.866	0.868	0.891	0.839	0.871	0.898	0.895	0.844
**Hematologic**	0.820	0.810	0.775	0.813	0.812	0.832	0.828	0.836	0.805	0.826	0.814	0.799	0.817	0.835	0.804	0.815	0.774
**Kidney**	0.825	0.811	0.787	0.812	0.798	0.840	0.819	0.809	0.793	0.808	0.810	0.812	0.825	0.822	0.837	0.817	0.803
**Liver**	0.814	0.755	0.767	0.746	0.810	0.838	0.733	0.806	0.780	0.813	0.735	0.834	0.699	0.823	0.810	0.815	0.677
**Lung**	0.786	0.797	0.769	0.800	0.789	0.799	0.809	0.807	0.785	0.805	0.787	0.800	0.795	0.798	0.792	0.807	0.748
**Ovarian**	0.790	0.783	0.769	0.792	0.793	0.811	0.777	0.804	0.766	0.800	0.792	0.790	0.786	0.801	0.789	0.791	0.734
**Pancreatic**	0.808	0.795	0.786	0.801	0.813	0.768	0.789	0.805	0.773	0.798	0.801	0.800	0.814	0.809	0.839	0.812	0.737
**Prostate**	0.657	0.658	0.657	0.647	0.676	0.650	0.682	0.688	0.688	0.695	0.639	0.678	0.675	0.652	0.689	0.669	0.608
**Sarcoma**	0.954	0.957	0.952	0.985	0.959	0.956	0.931	0.975	0.924	0.945	0.912	0.957	0.916	0.960	0.986	0.947	0.911
**Skin**	0.949	0.903	0.864	0.901	0.846	0.935	0.930	0.927	0.863	0.895	0.883	0.916	0.776	0.926	0.868	0.908	0.842
**Stomach**	1.000	1.000	1.000	1.000		1.000	1.000		1.000			1.000	1.000		1.000	1.000	1.000
**Uterine**	0.871	0.924	0.879	0.833	0.861	0.875	0.880	0.864	0.821	0.945	0.996	0.925	0.888	0.953	0.882	0.904	0.882
**Average**	0.851	0.850	0.833	0.846	0.836	0.856	0.851	0.851	0.834	0.852	0.839	0.861	0.839	0.852	0.859	0.858	0.809
**Rank**	9	10	16	11	14	4	7	8	15	6	13	1	12	5	2	3	17

(B)

ID	Tissue	Dataset	SurvExpress Variable	Samples	BLCA	BRCA	COADREAD	GBM	HNSC	KIRC	LAML	LUAD	LUSC	OV	UCEC	MULTI	CIN	LYM	MES	CIN70	PGC
146	Bladder	Dyrskjot Orntoft Bladder GSE5287	#CENSORED:SURVIVAL_MONTHS	30	1.000	1.000	1.000	1.000	1.000	1.000	1.000	1.000	1.000	1.000	1.000	1.000	1.000	1.000	1.000	1.000	0.986
134	Bladder	Leem Kim Bladder GSE13507	#CENSORED:SURVIVAL_OVERALL_MONTHS	165	0.853	0.872	0.799	0.874	0.843	0.876	0.865	0.876	0.832	0.840	0.845	0.837	0.886	0.820	0.857	0.925	0.819
404	Bladder	Riester Bladder GSE31684	#CENSORED:SURVIVAL_MONTHS	93	0.825	0.833	0.793	0.858	0.813	0.858		0.800	0.752	0.847		0.805	0.771	0.809	0.769	0.849	0.679
85	Bladder	Bladder Urothelial Carcinoma TCGA	#CENSORED:SURVIVAL_MONTHS	54	1.000	0.975	0.992	1.000		0.981	1.000	1.000	1.000	1.000	1.000	1.000	1.000	0.983	1.000	1.000	1.000
81	Bone	Buddingh Kuijjer Bone Survival GSE21257	#CENSORED:SURVIVAL.MONTHS	53	1.000	0.999	1.000	0.998	1.000	0.991	1.000	1.000	0.953	1.000	1.000	1.000	0.999	0.996	1.000	1.000	1.000
98	Brain	Nutt Louis Glioblastoma BROAD	#CENSORED:SURVIVAL_MONTHS	50	0.896	0.943	0.889	0.880	0.918	0.996	0.980	0.910	0.937	0.962	0.937	0.896	0.910	0.925	0.957	0.999	0.841
408	Brain	Joo Kim Jin Kim Seol Nam Glioblastoma GSE4266	#CENSORED:OVERALL_SURVIVAL_MONTHS	55	0.962	0.902	0.842	0.893	0.916	0.875	0.883	0.896	0.838	0.881	0.917	0.947	0.932	0.847	0.876	0.840	0.807
52	Brain	Lee Nelson Glioblastoma GSE13041 GPL96	#CENSORED:SURVIVAL_MONTHS	218	0.688	0.670	0.680	0.699	0.657	0.681	0.676	0.700	0.683	0.678	0.697	0.671	0.658	0.672	0.659	0.643	0.655
66	Brain	Deprez Turkheimer Glioblastoma GSE2817	#CENSORED:SURVIVAL_MONTHS	30	1.000	1.000	1.000	1.000	1.000	1.000	1.000	1.000	1.000	1.000	1.000	1.000	1.000	1.000	1.000	1.000	1.000
55	Brain	Philips Aldape Astrocitome GSE4271 GPL97	#CENSORED:SURVIVAL_MONTHS	77	0.708	0.665	0.735	0.670	0.731	0.608	0.658	0.706	0.730	0.722	0.680	0.652	0.690	0.643	0.698	0.702	0.590
56	Brain	Philips Aldape Astrocitome GSE4271 GPL96	#CENSORED:SURVIVAL_MONTHS	77	0.828	0.815	0.819	0.804	0.788	0.785	0.812	0.846	0.819	0.825	0.785	0.792	0.841	0.828	0.761	0.823	0.755
149	Brain	Lee Nelson Meningioma GSE16581	#CENSORED:SURVIVAL_MONTHS	67	1.000	1.000	1.000	1.000	1.000		1.000		0.988	1.000	1.000	0.998	1.000	1.000	1.000		1.000
57	Brain	Freije Nelson Glioblastoma GSE4412 GPL96	#CENSORED:SURVIVAL_MONTHS	85	0.842	0.822	0.793	0.871	0.811	0.793	0.784	0.854	0.835	0.834	0.848	0.850	0.842	0.796	0.813	0.818	0.791
58	Brain	Freije Nelson Glioblastoma GSE4412 GPL97	#CENSORED:SURVIVAL_MONTHS	85	0.688	0.724	0.736	0.674	0.681	0.708	0.655	0.669	0.787	0.744	0.734	0.664	0.676	0.735	0.743	0.764	0.677
77	Brain	Park Park Medulloblastoma Survival GSE30074	#CENSORED:SURVIVAL.MONTHS	30	1.000	1.000	1.000	1.000	1.000	1.000	1.000	1.000	1.000	1.000	1.000	1.000	1.000	1.000	1.000	1.000	0.992
205	Brain	Robinson Gilbertson Meduloblastoma GSE37418	#CENSORED:SURVIVAL_MONTHS	76	1.000	1.000	0.996	1.000	0.999	0.997	1.000	1.000	1.000	0.984	1.000	1.000	1.000	0.999	1.000	0.997	0.993
76	Brain	Remke Medulloblastoma GSE28245	#CENSORED:SURVIVAL.MONTHS	64	0.999	1.000	0.996	0.995	0.999	1.000	0.999	1.000	0.994	0.993	0.999	1.000	0.998	0.995	0.999	1.000	0.984
132	Brain	Murat Hegi Glioblastoma GSE7696	#CENSORED:SURVIVAL_MONTHS	84	0.785	0.782	0.744	0.756	0.761	0.815	0.768	0.772	0.772	0.826	0.750	0.815	0.754	0.801	0.801	0.807	0.734
97	Brain	Glioblastoma multiforme TCGA	#CENSORED:SURVIVAL_MONTHS	538	0.606	0.614	0.592	0.709	0.596	0.635	0.627	0.632	0.605	0.593	0.638	0.648	0.626	0.638	0.626	0.619	0.617
103	Brain	Brain Lower Grade Glioma TCGA	#CENSORED:SURVIVAL_MONTHS	110	0.912	0.944	0.926	0.893	0.918	0.915	0.889	0.907	0.884	0.869	0.906	0.910	0.922	0.878	0.924	0.919	0.855
164	Breast	Chin Gray Breast E-TABM-158	#CENSORED:SURVIVAL_MONTHS	117	0.821	0.791	0.833	0.921	0.855	0.853	0.848	0.910	0.800	0.879	0.805	0.933	0.876	0.881	0.862	0.854	0.783
401	Breast	Prat-Perou-Breast-GSE18229	#CENSORED:OFS	254	0.765	0.786	0.765	0.758	0.797	0.756	0.814	0.756	0.684	0.764	0.770	0.722	0.757	0.727	0.771	0.792	0.731
162	Breast	Bild Nevins Breast Survival GSE3143	#CENSORED:SURVIVL_MONHS	158	0.788	0.777	0.776	0.741	0.761	0.777	0.780	0.779	0.784	0.776	0.778	0.835	0.745	0.787	0.770	0.811	0.717
161	Breast	Ma Sgroi Breast GSE1378	#CENSORED:SURVIVAL_MONTHS	60	0.817	0.930	0.827	0.994	0.914	0.857	0.978	0.943	0.866	0.836	0.987	0.906	0.958	1.000	0.977	1.000	0.890
185	Breast	Enerly Yakhini Breast GSE19536	#CENSORED:SURVIVAL_MONTHS	111	0.811	0.841	0.815	0.778	0.826	0.906	0.877	0.851	0.829	0.906	0.867	0.888	0.859	0.841	0.851	0.877	0.779
155	Breast	Miller Bergh Breast GSE3494-GPL96	#CENSORED:SURVIVAL_MONTHS	236	0.738	0.735	0.762	0.733	0.738	0.732	0.726	0.794	0.736	0.780	0.795	0.771	0.738	0.709	0.727	0.738	0.727
156	Breast	Miller Bergh Breast GSE3494-GPL97	#CENSORED:SURVIVAL_MONTHS	236	0.608	0.610	0.637	0.656	0.654	0.612	0.590	0.650	0.695	0.651	0.663	0.601	0.622	0.661	0.665	0.659	0.693
157	Breast	Sgori Ma Breast GSE1379	#CENSORED:SURVIVAL_MONTHS	60	0.706	0.694	0.673	0.659	0.713	0.634	0.765	0.690	0.649	0.736	0.775	0.755	0.656	0.859	0.725	0.545	0.699
188	Breast	Staaf Borg Breast GSE25307	#CENSORED:SURVIVAL_MONTHS	551	0.567	0.574		0.615	0.546	0.554	0.589	0.542		0.520	0.499		0.633	0.547	0.557	0.626	0.562
158	Breast	Schmidt Gehrmann Breast GSE11121	#CENSORED:SURVIVAL_MONTHS	200	0.777	0.841	0.767	0.824	0.780	0.787	0.835	0.834	0.818	0.768	0.837	0.816	0.850	0.825	0.828	0.845	0.771
168	Breast	Vincent Darbon Breast GSE9893	#CENSORED:ORVERALL_SURVIVAL_MONTHS	155	0.914	0.899	0.861	0.849	0.904	0.893	0.896	0.938	0.870	0.912	0.894	0.947	0.903	0.921	0.883	0.913	0.867
127	Breast	Kao Huang Breast GSE20685	#CENSORED:SURVIVAL_MONTHS	327	0.747	0.760	0.738	0.747	0.793		0.742		0.745	0.776	0.743	0.745	0.727	0.765	0.769		0.673
86	Breast	Breast Invasive Carcinoma TCGA	#CENSORED:SURVIVAL_MONTHS	502	0.710	0.900	0.761	0.741	0.708	0.805	0.737	0.741	0.679	0.797	0.730	0.805	0.775	0.779	0.723	0.733	0.741
395	Colon	Sveen-Agesen-Colon-GSE24551-GPL5175	#CENSORED:SURVIVAL	243	0.742	0.727	0.750	0.730	0.738	0.707	0.719	0.760	0.716	0.744	0.757	0.737	0.743	0.727	0.756	0.733	0.661
396	Colon	Sveen-Skotheim-Colon-GSE24549-GPL5175	#CENSORED:DISEASE.FREE.SURVIVAL	83	0.612	0.611	0.561	0.557	0.626	0.552	0.565	0.628	0.672	0.609	0.583	0.607	0.674	0.646	0.641	0.663	0.556
397	Colon	Sveen-Skotheim-Colon-GSE24549-GPL11028	#CENSORED:DISEASE_FREE_SURVIVAL	83	0.834	0.832	0.728	0.824	0.772	0.836	0.827	0.792	0.809	0.835	0.821	0.758	0.807	0.812	0.883	0.847	0.715
398	Colon	Sveen-Agesen-Colon-GSE24551-GPL11028	#CENSORED:DISEASE_FREE_SURVIVAL_YEARS	160	0.754	0.743	0.705	0.760	0.733	0.739	0.761	0.754	0.747	0.771	0.733	0.763	0.747	0.732	0.772	0.790	0.692
36	Colon	Sveen Colon (TrainSet) GSE24550	#CENSORED:Survival_Months	77	1.000	0.985	0.992	1.000	0.999	0.990	0.999	0.991	0.984	1.000		0.994	1.000	0.999	0.997	1.000	0.885
34	Colon	Smith Beauchamp Colon GSE17536	#CENSORED:SURVIVAL_MONTHS	177	0.765	0.750	0.752	0.743	0.732	0.738	0.749	0.777	0.726	0.748	0.772	0.766	0.729	0.769	0.781	0.745	0.706
32	Colon	Staub Colon GSE12945	#CENSORED:Survival_Months	62	1.000	0.998	0.998	0.998	1.000	0.998	1.000	1.000	1.000	1.000	1.000	1.000	1.000	1.000	1.000	0.998	1.000
384	Colon	Reid Pierotti Colon GSE16125 GPL5175	#CENSORED:SURVIVAL_YEARS	32	0.704	0.716	0.782	0.514	0.796	0.810	0.768	0.798	0.789	0.676	0.669	0.758	0.894	0.871	0.770	0.711	0.702
383	Colon	Loboda Yeatman Colon GSE28722	#CENSORED:OVERALL.SURVIVAL	125	0.797	0.833	0.771	0.761	0.757	0.795	0.785	0.852	0.742	0.804	0.811	0.820	0.787	0.775	0.803	0.791	0.809
394	Colon	Sheffer-Domany-Colon-GSE41258	#CENSORED:SURVIVAL	243	0.742	0.727	0.750	0.730	0.738	0.707	0.719	0.760	0.716	0.744	0.757	0.737	0.743	0.727	0.756	0.733	0.661
47	Colon	Sveen Colon (TestSet) GSE30378	#CENSORED:Survival_Months	83	0.834	0.832	0.728	0.824	0.772	0.836	0.827	0.792	0.809	0.835	0.821	0.758	0.807	0.812	0.883	0.847	0.715
96	Colon	Colon Rectal Adenocarcinoma TCGA	#CENSORED:SURVIVAL_MONTHS	151	0.991	0.913	1.000	0.973	0.950	1.000	0.853	0.899	0.957	0.998	0.952	1.000	0.984	0.998	0.967	0.889	0.935
89	Esophagus	Rao Giddings Esophagus GSE11595	#CENSORED:SURVIVAL.MONTHS	34	0.939	0.998	0.996	1.000	0.926	1.000	0.996	1.000	1.000	1.000	1.000	1.000	1.000	1.000	1.000	1.000	0.953
90	Esophagus	Peters C.Fitzgerald Esophagus GSE19417	#CENSORED:SURVIVAL.MONTHS	70	0.761	0.773	0.692	0.744	0.779	0.781	0.761	0.789	0.734	0.829	0.700	0.802	0.810	0.752	0.775	0.756	0.694
211	Head and Neck	Chung Perou Survival Head and Neck Cancer GSE	#CENSORED:SURVIVAL.EVENT.MONTHS	71	1.000	0.997	0.999	0.999	0.942	1.000	0.988	0.963	0.988	0.940	0.985	1.000	0.879	0.995	0.997	0.999	0.942
212	Head and Neck	Saintigny Mao Survival Oral cancer GSE26549	#CENSORED:ORAL.CANCER.FREE.SURVIVAL.TIM	86	0.869	0.836	0.853	0.919	0.912	0.929	0.934	0.934	0.912	0.908	0.964	0.918	0.928	0.897	0.980	0.939	0.899
100	Head and Neck	Head and Neck squamous cell carcinoma TCGA	#CENSORED:SURVIVAL_MONTHS	283	0.672	0.747	0.688	0.738	0.836	0.722	0.714	0.744	0.737	0.751	0.655	0.755	0.709	0.721	0.718	0.748	0.691
136	Hematologic	Herold Bohlander CLL GSE22762 GPL97	#CENSORED:SURVIVAL.MONTHS	30	0.878	0.821	0.789	0.774	0.860	0.853	0.803	0.961	0.914	0.821	0.925	0.803	0.720	0.864	0.900	0.674	0.871
138	Hematologic	Herold Bohlander CLL GSE22762 GPL570	#CENSORED:SURVIVAL.MONTHS	70	0.984	0.963	0.950	0.984	0.970	0.997	0.963	0.992	0.906	0.996	0.951	0.886	0.965	0.983	0.999	0.980	0.859
143	Hematologic	Metzeler Buske CM-AML GSE12417-GPL570	#CENSORED:SURVIVAL_MONTHS	79	0.877	0.813	0.826	0.877	0.817	0.818	0.869	0.888	0.860	0.888	0.880	0.870	0.815	0.817	0.841	0.833	0.763
147	Hematologic	Shaughnessy Multiple Myeloma GSE2658	#CENSORED:SURVIVAL_MONTHS	559	0.721	0.711	0.671	0.728	0.718	0.732	0.683	0.719	0.718	0.734	0.694	0.733	0.689	0.702	0.706	0.723	0.632
171	Hematologic	Rosenwald Staudt DLBC Lymph	#CENSORED:Survival_Months	240	0.579	0.549	0.561	0.520	0.518	0.594	0.573	0.599	0.556	0.603	0.587	0.574	0.627	0.596	0.590	0.604	0.513
140	Hematologic	Metzeler Buske CM-AML GSE12417-GPL97	#CENSORED:SURVIVAL.MONTHS	163	0.619	0.616	0.611	0.587	0.653	0.557	0.599	0.592	0.642	0.605	0.582	0.630	0.604	0.635	0.618	0.633	0.612
499	Hematologic	Bullinger-Pollack AML GSE425-GPL318	#CENSORED:SURVIVAL_MONTHS	44	1.000	0.996	0.867	0.995	0.950	1.000	1.000	1.000	0.918	1.000	1.000	0.855	1.000	1.000	1.000		0.997
500	Hematologic	Bullinger-Pollack AML GSE425-GPL319	#CENSORED:SURVIVAL_MONTHS	49	0.999	0.959	0.791	0.989	1.000	0.997	0.993	0.987	0.951	0.961	0.921	0.871	0.998	0.998	0.990	1.000	0.994
170	Hematologic	Rosenwald Matle Cell Lymph	#CENSORED:SURVIVAL_YEARS	92	0.763	0.740	0.763	0.783	0.691	0.782	0.782	0.767	0.752	0.729	0.704	0.762	0.787	0.775	0.627	0.798	0.656
388	Hematologic	Jais Leukemia	#CENSORED:SURVIVAL.EVENT.YEARS	53	0.957	0.903	0.922	0.998	1.000	0.979	0.984	0.859	0.881	0.948	0.929	0.994	0.954	0.998	1.000	1.000	0.874
169	Hematologic	Rosenwald Folicular Lymph	#CENSORED:SURVIVAL_MONTHS	187	0.730	0.700	0.676	0.726	0.719	0.711	0.691	0.721	0.703	0.708	0.694	0.719	0.675	0.721	0.671	0.680	0.705
167	Hematologic	Alizadeh Staudt DLB Lymph GSE60	#CENSORED:SURVIVAL.MONTHS	34	0.728	0.922	0.819	0.795	0.856	1.000	0.929	0.985	0.836	0.884	0.834	0.765	0.998	1.000	0.681	1.000	0.711
91	Hematologic	Geng Lymphoma GSE23501	#CENSORED:OVERALL.SURVIVAL	69	0.999	1.000	1.000	0.997	1.000	1.000	1.000	1.000	0.978	0.996	1.000	1.000	0.937	1.000	0.999	0.992	0.994
129	Hematologic	Metzeler Buske AML GSE12417-GPL96	#CENSORED:SURVIVAL.EVENT	163	0.688	0.705	0.650	0.683	0.694	0.722	0.702	0.691	0.707	0.748	0.754	0.746	0.708	0.684	0.727	0.738	0.675
102	Hematologic	Acute Myeloid Leukemia TCGA	#CENSORED:SURVIVAL_MONTHS	168	0.775	0.752	0.734	0.762	0.741	0.735	0.847	0.784	0.749	0.764	0.756	0.777	0.776	0.747	0.719	0.763	0.749
70	Kidney	Zhao Renal Kidney GSE3538	#CENSORED:SURVIVAL.MONTHS	177	0.720	0.684	0.683	0.701	0.677	0.740	0.715	0.692	0.658	0.688	0.677	0.699	0.761	0.717	0.738	0.713	0.701
101	Kidney	Kidney renal clear cell carcinoma TCGA	#CENSORED:SURVIVAL_MONTHS	468	0.755	0.748	0.716	0.734	0.719	0.780	0.744	0.735	0.721	0.736	0.754	0.738	0.713	0.749	0.774	0.739	0.707
525	Kidney	Fergelot Renal Kidney GSE29609	#CENSORED:SURVIVAL_MONTHS	39	1.000	1.000	0.963	1.000	1.000	1.000	1.000	1.000	1.000	1.000	1.000	1.000	1.000	1.000	1.000	1.000	1.000
74	Liver	Hoshida Golub Liver GSE10143	#CENSORED:SURVIVAL.MONTHS	162	0.736	0.697	0.707	0.706	0.747	0.768	0.688	0.718	0.711	0.737	0.733	0.774	0.675	0.752	0.785	0.733	0.630
83	Liver	Hoshida Golub Liver GSE10186	#CENSORED:SURVIVAL.MONTHS	80	0.891	0.814	0.827	0.786	0.872	0.908	0.777	0.894	0.849	0.890	0.738	0.894	0.722	0.894	0.835	0.897	0.724
14	Lung	Zhu Tsao Lung GSE14814	#CENSORED:Overall.Survival.Months	90	0.707	0.760	0.761	0.743	0.731	0.708	0.731	0.769	0.719	0.730	0.721	0.718	0.740	0.710	0.750	0.759	0.730
14	Lung	Zhu Tsao Lung GSE14814	#CENSORED:Overall.Survival.Months	90	0.875	0.865	0.856	0.952	0.891	0.881	0.878	0.881	0.852	0.896	0.893	0.856	0.905	0.920	0.857	0.952	0.842
130	Lung	Wilkerson Lung GSE17710	#CENSORED:Survival_Months	56	0.883	0.995	0.842	0.970	0.993	0.802	0.989	0.982	0.909	0.956	0.956	0.960	0.922	0.983	0.988	0.909	0.937
16	Lung	Chen Lung GSE4882	#CENSORED:Survival.Months	125	0.619	0.602	0.499	0.589	0.560	0.659	0.603	0.655	0.583	0.628	0.615	0.513	0.604	0.507	0.574	0.567	0.497
17	Lung	Hou Philipsen Lung GSE19188	#CENSORED:Survival_data	64	0.839	0.866	0.870	0.928	0.873	0.855	0.927	0.881	0.763	0.889	0.830	0.832	0.905	0.885	0.854	0.856	0.765
65	Lung	Tomida Lung GSE13213	#CENSORED:Survival_Months	117	0.793	0.831	0.822	0.843	0.797	0.851	0.870	0.827	0.833	0.812	0.824	0.800	0.855	0.835	0.805	0.869	0.785
69	Lung	Directors Challenge Consortium NCI Lung	#CENSORED:Survival_Months	444	0.675	0.673	0.626	0.661	0.669	0.683	0.671	0.644	0.641	0.685	0.671	0.668	0.642	0.664	0.655	0.666	0.657
392	Lung	Rousseaux GSE30219	#CENSORED:Survival_Months	264	0.713	0.721	0.688	0.732	0.714	0.734	0.702	0.714	0.714	0.677	0.680	0.724	0.721	0.707	0.701	0.700	0.678
12	Lung	Tomida Lung Cancer Survival GSE4716	#CENSORED:Survival_Months	50	0.876	0.784	0.838	0.768	0.775	1.000	0.984	0.890	0.848	0.990	0.791	0.921	0.883	0.994	0.929	1.000	0.818
15	Lung	Baty Lung GSE11117	#CENSORED:SURVIVAL.MONTHS	41	1.000	1.000	1.000	0.971	0.998	1.000	1.000	0.998	1.000	1.000	1.000	1.000	1.000	1.000	0.992	1.000	0.946
139	Lung	Roepman Lung	#CENSORED:Survival_Months	148	0.695	0.710	0.702	0.720	0.675	0.750	0.661	0.737	0.700	0.698	0.742	0.675	0.682	0.697	0.743	0.700	0.674
115	Lung	Chitale Lung	#CENSORED:Survival_Months	185	0.881	0.872	0.833	0.873	0.883	0.860	0.871	0.819	0.858	0.843	0.847	0.860	0.894	0.878	0.846	0.886	0.832
105	Lung	Lung Adenocarcinoma TCGA	#CENSORED:SURVIVAL_MONTHS	255	0.763	0.791	0.731	0.772	0.780	0.791	0.772	0.887	0.731	0.756	0.786	0.814	0.738	0.729	0.762	0.733	0.743
106	Lung	Lung Squamous Cell Carcinoma TCGA	#CENSORED:SURVIVAL_MONTHS	205	0.724	0.732	0.684	0.736	0.721	0.742	0.713	0.697	0.870	0.750	0.731	0.773	0.732	0.798	0.713	0.685	0.694
11	Lung	Bild Nevins Lung GSE3141	#CENSORED:SURVIVAL_MON	108	0.765	0.820	0.779	0.819	0.756	0.797	0.731	0.758	0.768	0.785	0.746	0.801	0.763	0.765	0.769	0.785	0.679
10	Lung	Raponi Beer Lung GSE4573	#CENSORED:Survival_Months	130	0.659	0.639	0.621	0.731	0.671	0.639	0.736	0.720	0.643	0.704	0.604	0.708	0.691	0.724	0.626	0.779	0.649
5	Lung	Larsen Lung GSE5123	#CENSORED:Survival_Months	51	0.997	0.996	0.913	0.893	1.000	1.000	1.000	0.970	0.992	0.993	1.000	1.000	0.996	0.885	0.992	1.000	0.848
391	Lung	Botling GSE37745	#CENSORED:Survival_Months	196	0.644	0.668	0.666	0.681	0.704	0.652	0.651	0.679	0.649	0.680	0.675	0.693	0.670	0.683	0.693	0.682	0.631
131	Lung	Okayama Kohno Lung GSE31210	#CENSORED:Survival_Months	226	0.851	0.826	0.886	0.831	0.858	0.835	0.881	0.855	0.870	0.860	0.879	0.899	0.813	0.860	0.843	0.871	0.828
154	Lung	Tang Xiao Lung GSE42127	#CENSORED:Survival_Months	176	0.771	0.781	0.763	0.795	0.736	0.745	0.806	0.768	0.757	0.774	0.753	0.792	0.737	0.741	0.757	0.733	0.726
184	Miscellaneous	Savola Suvi Ewing sarcoma GSE17679	#CENSORED:OVS_TIME	88	0.954	0.957	0.952	0.985	0.959	0.956	0.931	0.975	0.924	0.945	0.912	0.957	0.916	0.960	0.986	0.947	0.911
38	Ovarian	Mok Birrer Ovarian GSE18520	#CENSORED:SURVIVAL_MONTHS	53	0.871	0.906	0.877	0.949	0.946	0.959	0.925	0.935	0.847	0.848	0.878	0.907	0.874	0.846	0.920	0.872	0.814
40	Ovarian	Spentzos Konstantinopoulos Ovarian GSE19161	#CENSORED:SURVIVAL_MONTHS	61	0.504	0.546	0.509	0.533	0.553	0.684	0.554	0.607	0.568	0.550	0.603	0.642	0.547	0.613	0.638	0.588	0.514
367	Ovarian	Fountzilas Konstantinopoulos Ovarian GSE19829-	#CENSORED:OVERALL_SURVIVAL_MONTHS	42	1.000	0.995	0.940	0.990	1.000	1.000	1.000	0.998	1.000	1.000	0.942	1.000	1.000	1.000	1.000	1.000	0.872
402	Ovarian	Spentzos Ovarian GSE31245	#CENSORED:OVERALL_SURVIVAL_MONTH	58	0.951	0.899	0.925	0.966	1.000	1.000	0.989	0.966	0.879	0.997	0.946	0.985	0.938	0.910	0.943	0.975	0.864
39	Ovarian	Crijns van der Ze Ovarian GSE13876	#CENSORED:SURVIVAL_MONTHS	415	0.765	0.746	0.761	0.760	0.749	0.761	0.706	0.774	0.800	0.724	0.739	0.700	0.751	0.759	0.802	0.820	0.660
41	Ovarian	Lage Denkert Ovarian GSE14764	#CENSORED:SURVIVAL_MONTHS	80	0.789	0.728	0.736	0.798	0.773	0.720	0.806	0.815	0.763	0.815	0.839	0.779	0.838	0.788	0.832	0.759	0.686
369	Ovarian	Ferriss Lee Ovarian GSE30161	#CENSORED:OVERALL_SURVIVAL_MONTHS	58	0.984	0.895	0.930	0.859	0.955	0.990	0.885	0.939	0.846	0.917	0.967	0.861	0.913	0.955	0.938	0.935	0.737
46	Ovarian	Yoshihara Tanaka Ovarian GSE17260	#CENSORED:SURVIVAL.MONTHS	110	0.802	0.769	0.735	0.785	0.774	0.781	0.796	0.865	0.746	0.761	0.770	0.796	0.841	0.788	0.781	0.802	0.744
370	Ovarian	Japanese Serous Ovarian Cancer Study Group GS	#CENSORED:SURVIVAL_MONTHS	40	1.000	1.000	1.000	1.000	1.000	1.000	1.000	1.000	1.000	1.000	1.000	1.000	1.000	1.000	1.000	1.000	0.997
37	Ovarian	Yoshihara Tanaka Ovarian GSE32062	#CENSORED:SURVIVAL_MONTHS	255	0.690	0.700	0.699	0.695	0.683	0.703	0.689	0.700	0.702	0.705	0.703	0.680	0.670	0.698	0.645	0.681	0.632
368	Ovarian	Gillete Gottesman Ovarian GSE30009	#CENSORED:SURVIVAL_MONTHS	103	0.573	0.644	0.612	0.646	0.623	0.640	0.598	0.555	0.575	0.601	0.613	0.567	0.526		0.559	0.623	
375	Ovarian	Ovarian serous cystadenocarcinoma TCGA	#CENSORED:SURVIVAL_MONTHS	578	0.636	0.652	0.656	0.622	0.627	0.624	0.633	0.646	0.627	0.732	0.648	0.663	0.633	0.663	0.615	0.629	0.631
35	Ovarian	Tothill Bowtell Survival Ovarian GSE9891	#CENSORED:SURVIVAL_MONTHS	285	0.761	0.711	0.708	0.742	0.735	0.732	0.663	0.729	0.707	0.770	0.732	0.728	0.747	0.690	0.753	0.687	0.673
42	Ovarian	Nevins Bild Ovarian GSE3149	#CENSORED:SURVIVAL_MONTHS	124	0.817	0.825	0.796	0.841	0.801	0.850	0.739	0.798	0.760	0.846	0.798	0.843	0.821	0.857	0.742	0.764	0.787
43	Ovarian	Bonome Birrer Ovarian GSE26712	#CENSORED:SURVIVAL_MONTHS	185	0.701	0.726	0.658	0.690	0.681	0.712	0.665	0.737	0.676	0.730	0.708	0.694	0.699	0.646	0.672	0.727	0.659
62	Pancreatic	Zhang Hussain Pancreatic GSE28735	#CENSORED:SURVIVAL_MONTHS	84	0.848	0.807	0.762	0.822	0.854	0.792	0.807	0.823	0.773	0.787	0.790	0.837	0.838	0.809	0.882	0.866	0.702
61	Pancreatic	Stratford Yeh Pancreatic GSE21501	#CENSORED:SURVIVAL_MONTHS	102	0.768	0.783	0.809	0.780	0.772	0.743	0.771	0.788	0.773	0.809	0.812	0.763	0.789	0.809	0.796	0.757	0.773
114	Prostate	Sboner Rubin Prostate GSE16560	#CENSORED:SURVIVAL_MONTH	281	0.657	0.658	0.657	0.647	0.676	0.650	0.682	0.688	0.688	0.695	0.639	0.678	0.675	0.652	0.689	0.669	0.608
78	Skin	Jonsson Skin Survival GSE22153	#CENSORED:SURVIVAL.MONTHS	54	0.949	0.903	0.864	0.901	0.846	0.935	0.930	0.927	0.863	0.895	0.883	0.916	0.776	0.926	0.868	0.908	0.842
112	Stomach	Stomach Adenocarcinoma TCGA	#CENSORED:SURVIVAL_MONTHS	57	1.000	1.000	1.000	1.000		1.000	1.000		1.000			1.000	1.000		1.000	1.000	1.000
110	Uterine	Uterine Corpus Endometrioid Carcinoma TCGA	#CENSORED:SURVIVAL_MONTHS	332	0.871	0.924	0.879	0.833	0.861	0.875	0.880	0.864	0.821	0.945	0.996	0.925	0.888	0.953	0.882	0.904	0.882
				AVERAGE	0.816	0.817	0.799	0.816	0.812	0.824	0.819	0.826	0.808	0.824	0.813	0.823	0.817	0.826	0.822	0.824	0.774
				RANK	11	10	16	12	14	5	8	1	15	3	13	6	9	2	7	4	17

Supplementary Table 5. Comparison of our network clinical association (NCA) algorithm with other previously reported algorithms.

Reference	Univariate Association	Multivariate Association	Network	Exploration Criteria	Finish Criteria
THIS WORK: Network Clinical Association (NCA)	Cox	Cox	PPI	Top K% (K=5) of the improved	Until no improvements
Chuang HY, Lee E, Liu YT, Lee D, Ideker T (2007) Network-based classification of breast cancer metastasis. Mol Syst Biol 3: 140.	Z-Score & Classification	Sum of Z scores and Mutual Information Classifier	PPI	The best	d+1 nodes maximum, or no improvement
Li J, Roebuck P, Grunewald S, Liang H (2012) SurvNet: a web server for identifying network-based biomarkers that most correlate with patient survival data. Nucleic Acids Res 40: W123-126.	Z-score of Cox p-value. Then filtering.	Normalized z-score average	PPI	All improved	d nodes maximum or no improvement
Wu G, Stein L (2012) A network module-based method for identifying cancer prognostic signatures. Genome Biol 13: R112.	Cox	(Uses univariate Cox to module averages)	Connections from diverse network databases. Edged weighted on gene expression correlation.	Markov Clustering to generate modules. Modules are averaged and chosen depend on the linear model of a Cox-supervised principal component analysis.	(Run once)
Winter C, Kristiansen G, Kersting S, Roy J, Aust D, et al. (2012) Google goes cancer: improving outcome prediction for cancer patients by network-based ranking of marker genes. PLoS Comput Biol 8: e1002511.	Classiffier and Cross-Validation	Classiffier and Cross-Validation	HPRD & CoExpressDB weighted by NetRank (Google-Like)	Forward Selection is ran for all genes, then chooses the best model.	(Run once)
Li J, Lenferink AE, Deng Y, Collins C, Cui Q, et al. (2010) Identification of high-quality cancer prognostic markers and metastasis network modules. Nat Commun 1: 34.	Classification	Weighted Sum	Gene Ontology	Forward Selection using Shrunken Centroids is ran for all genes then chooses the best model.	(Run once)

**COLUMNS:**	**DESCRIPTION:**
**Univariate Association**	Specify the initial association of genes to outcome
**Multivariate Association**	Specify the association of genes to outcome once they are agglomerated in larger modules
**Network**	Specify the network used or how it was built
**Exploration Criteria**	Criteria used to grow, expand, or select genes
**Finish Criteria**	Criteria used to finish the procedure

PPI - Protein-Protein Interaction Network

Supplementary Table 6. Mutations (A) and copy number variations (B) of genes in the multi-NCA biomarker.

(A)

	BLCA		BRCA		COADREAD		GBM		HNSC		KIRC		LAML		LUAD		LUSC		OV		UCEC	
**Samples**	26	27	239	240	24	38	138	99	137	139	202	187	76	74	52	56	84	81	166	148	114	105
**Gene**	**High**	**Low**	**High**	**Low**	**High**	**Low**	**High**	**Low**	**High**	**Low**	**High**	**Low**	**High**	**Low**	**High**	**Low**	**High**	**Low**	**High**	**Low**	**High**	**Low**
ACVR1B	0	0	2	2	0	2	0	0	3	0	3	1	0	0	0	3	1	1	1	0	4	3
AKT2	0	0	1	1	1	0	0	0	1	3	1	2	0	0	0	1	2	0	0	0	3	2
BCL3	0	0	0	1	0	1	0	0	0	0	0	1	0	0	0	0	0	0	1	1	1	0
C2	1	1	1	0	0	0	2	0	0	1	0	1	1	0	3	1	2	1	0	1	2	0
C3	0	1	2	1	0	1	0	5	5	2	4	1	1	0	2	5	3	2	0	1	8	4
CALR	0	0	0	1	0	0	0	0	0	2	1	0	0	2	0	0	1	1	0	1	3	1
CCNH	0	2	0	0	0	1	0	0	0	0	0	1	0	0	0	0	0	0	0	1	2	2
CFTR	0	0	3	2	0	1	1	1	3	3	2	2	0	0	4	0	3	4	1	1	6	6
CIITA	0	1	0	1	0	2	1	1	4	3	0	2	0	0	1	2	0	3	0	1	4	5
CTGF	0	1	0	2	0	0	0	0	0	1	1	0	0	0	1	1	0	1	0	0	4	1
DDX5	0	1	0	1	0	0	1	1	0	0	1	0	0	0	1	0	0	1	1	1	1	4
DUT	0	0	0	0	0	0	0	0	0	0	0	0	0	0	0	0	0	0	0	0	0	0
ESR1	0	0	1	1	1	1	1	1	0	2	1	0	0	0	0	1	4	3	0	0	6	5
HMGN1	0	1	0	0	0	0	0	0	0	0	0	0	0	0	0	0	0	0	0	0	1	0
HRAS	0	2	0	0	0	0	0	0	4	6	0	1	0	0	1	0	1	4	0	0	1	0
ITGA4	2	3	0	0	0	1	1	1	3	1	0	0	0	0	2	2	3	1	0	3	4	4
ITSN1	2	3	2	1	1	1	1	2	2	4	2	0	0	0	5	8	2	2	0	3	7	4
JUN	0	0	0	1	0	0	0	0	0	0	1	0	0	0	1	0	2	1	0	0	0	1
KCNB1	0	0	1	1	0	2	2	0	2	1	0	0	0	0	3	14	0	0	0	0	5	0
KCNJ12	0	0	1	2	0	1	2	0	6	2	7	2	0	0	3	3	1	5	2	1	0	3
LGALS8	0	0	0	1	0	0	2	1	0	0	0	1	0	0	0	0	0	0	1	0	2	1
LMO4	1	0	0	1	0	0	0	0	1	0	2	1	0	0	1	0	0	0	0	0	1	1
LRP1	4	4	3	5	1	3	3	5	9	7	4	6	0	0	4	6	8	5	4	2	10	14
LRPAP1	0	0	0	0	0	0	0	0	0	1	0	0	0	0	0	0	0	0	0	1	0	3
MAP2	0	2	6	1	1	5	0	1	7	5	1	0	0	2	1	6	3	4	4	3	5	5
MARK4	0	1	1	0	0	1	0	0	3	0	0	1	0	0	2	0	0	3	0	2	2	2
MLL2	3	8	3	4	0	0	3	2	26	23	4	7	0	1	4	9	15	18	2	0	11	16
MMP15	1	0	2	0	0	1	0	0	2	0	0	0	0	0	0	0	0	1	1	0	0	2
MTCH2	0	0	0	0	0	0	0	0	1	0	2	0	0	0	0	0	0	0	0	0	2	1
NEDD9	0	1	3	3	0	1	0	0	1	1	0	2	0	0	1	0	1	1	0	0	3	3
PDC	0	0	0	0	0	1	1	0	1	0	1	0	0	1	0	1	0	0	0	0	0	1
PDIA3	1	0	0	0	0	1	0	1	0	0	0	0	0	0	0	0	0	1	0	2	1	2
PRKACA	0	0	1	1	0	0	0	0	3	1	0	1	0	0	0	0	0	0	0	0	4	3
PTPN7	0	1	1	1	0	1	0	0	2	0	1	0	0	0	0	0	0	1	0	0	1	2
RPS6KA1	0	1	1	0	0	1	0	0	1	3	0	2	0	0	0	1	1	1	0	0	1	1
RRAD	1	0	0	1	0	1	1	1	0	0	2	1	0	0	2	0	0	0	0	0	0	3
SLC9A6	0	0	0	0	1	1	0	1	3	0	0	0	0	0	3	2	2	0	0	1	3	4
SMAD2	0	1	3	0	0	1	0	0	1	2	1	1	0	0	1	1	0	1	0	0	3	5
SNAP25	0	1	1	0	0	0	0	0	0	1	0	0	0	0	1	1	1	0	0	0	4	3
TH	0	0	0	0	0	0	0	1	0	2	1	0	0	0	1	1	0	2	0	0	1	1
TOP1	0	1	1	0	0	0	1	0	1	1	2	1	0	0	1	1	0	2	0	1	6	3

(B)

Cancer Type	BLCA				BRCA				COADREAD				GBM				HNSC				KIRC				LAML				LUAD				LUSC				OV				UCEC			
Multi-Cancer Risk	High Risk		Low Risk		High Risk		Low Risk		High Risk		Low Risk		High Risk		Low Risk		High Risk		Low Risk		High Risk		Low Risk		High Risk		Low Risk		High Risk		Low Risk		High Risk		Low Risk		High Risk		Low Risk		High Risk		Low Risk	
Samples	27		26		232		238		73		72		248		240		137		139		209		199		73		75		89		107		84		81		261		235		131		126	
Gene	Losses	Gains	Losses	Gains	Losses	Gains	Losses	Gains	Losses	Gains	Losses	Gains	Losses	Gains	Losses	Gains	Losses	Gains	Losses	Gains	Losses	Gains	Losses	Gains	Losses	Gains	Losses	Gains	Losses	Gains	Losses	Gains	Losses	Gains	Losses	Gains	Losses	Gains	Losses	Gains	Losses	Gains	Losses	Gains
ACVR1B	0	0	0	3	1	1	0	1	0	0	2	0	0	3	1	1	0	1	0	0	0	0	0	0	0	0	0	0	0	1	0	2	0	0	0	0	0	3	2	4	0	1	0	0
AKT2	0	2	0	1	0	1	0	6	0	0	0	0	0	0	4	0	0	2	0	2	0	0	0	0	0	0	0	0	0	0	0	1	0	5	0	3	1	24	1	14	0	2	0	3
BCL3	0	0	0	0	0	3	0	3	0	0	0	0	1	1	4	0	0	1	0	2	0	1	0	0	0	0	0	0	0	4	0	4	0	2	0	0	1	15	1	1	0	2	0	0
C2	0	1	0	0	0	0	0	0	0	0	0	0	0	0	0	0	0	3	1	1	0	0	0	0	0	0	0	0	0	3	0	4	0	0	0	1	0	11	0	9	0	1	0	0
C3	0	0	0	0	0	1	0	3	0	0	0	0	0	0	0	0	1	0	1	0	1	0	0	0	0	0	0	0	1	0	1	0	1	0	0	1	1	3	0	2	0	1	1	0
CALR	0	0	0	0	0	2	0	4	0	0	0	0	0	0	0	3	1	1	0	1	0	0	0	0	0	0	0	0	1	0	1	0	1	1	0	1	0	40	0	21	0	1	0	2
CCNH	0	0	0	0	0	0	1	0	1	0	0	0	0	0	1	0	1	0	1	0	0	0	0	0	0	0	0	0	2	0	1	0	0	0	1	0	8	0	8	0	0	0	0	0
CFTR	0	0	0	0	0	0	0	0	0	0	0	1	0	11	0	24	0	0	0	2	0	1	0	1	0	0	0	0	0	4	1	1	0	2	0	1	1	7	0	5	0	0	0	2
CIITA	0	1	0	0	0	10	0	1	0	0	0	0	0	2	1	0	0	1	0	1	0	0	0	0	0	0	0	0	0	3	0	0	0	0	0	0	1	6	1	1	0	0	0	1
CTGF	0	1	0	0	0	0	0	4	0	0	0	0	2	0	5	0	1	1	1	1	1	0	0	0	0	0	0	0	1	0	2	0	0	0	0	0	1	1	1	1	0	1	0	1
DDX5	0	1	0	0	0	20	0	14	0	1	0	1	0	0	0	1	0	1	0	1	0	0	0	0	0	0	0	0	0	1	0	4	0	1	0	0	0	10	0	2	0	1	0	1
DUT	0	1	0	0	0	1	1	1	0	0	1	0	1	0	1	0	0	0	1	0	0	0	0	0	0	0	0	0	4	1	2	0	0	0	0	0	3	1	6	1	0	0	0	0
ESR1	0	0	0	0	0	2	1	6	0	0	0	0	2	0	5	0	0	0	0	0	1	0	0	0	0	0	0	0	1	0	4	0	0	0	0	1	1	5	3	5	0	0	0	0
HMGN1	0	0	0	1	0	3	0	0	0	0	0	0	0	0	1	0	0	0	0	1	0	0	0	0	0	0	0	0	0	2	1	0	0	0	0	0	1	2	1	3	0	0	0	1
HRAS	0	0	0	0	0	1	1	1	0	0	0	0	1	0	8	0	2	0	0	1	0	1	0	0	0	0	0	0	0	0	0	0	0	0	0	0	2	3	1	1	2	0	0	0
ITGA4	0	0	0	0	0	0	0	0	0	0	0	0	0	0	0	1	0	4	0	2	0	1	0	1	0	0	0	0	0	2	0	1	0	1	0	2	0	5	0	7	0	0	0	0
ITSN1	0	0	0	0	0	0	0	0	0	0	0	0	0	0	1	0	0	1	0	1	0	0	0	0	0	0	0	0	0	1	0	0	0	0	0	0	0	3	0	4	0	0	0	0
JUN	0	0	0	0	0	1	2	2	0	0	0	0	1	0	0	0	0	1	0	1	0	0	0	0	0	0	0	0	0	2	1	2	0	0	0	0	2	5	2	8	0	0	0	0
KCNB1	0	0	0	0	0	14	0	3	0	4	0	3	0	3	0	2	0	0	1	0	0	1	0	0	0	0	0	0	1	2	0	2	0	2	0	1	0	17	0	8	0	2	0	0
KCNJ12	0	0	0	1	1	5	2	0	0	0	0	0	0	0	1	1	2	0	0	1	0	0	0	0	0	0	0	0	1	0	1	0	1	1	0	2	4	4	6	1	0	1	0	1
LGALS8	0	1	0	0	0	13	0	13	1	0	0	0	0	5	0	0	0	1	0	2	0	0	0	0	0	0	0	0	0	4	0	8	0	2	0	2	0	14	0	12	0	2	0	1
LMO4	1	1	0	1	1	0	1	1	0	0	0	0	0	1	0	1	0	0	0	1	0	0	0	0	0	0	0	0	1	0	1	1	0	1	0	0	1	3	1	1	0	0	0	0
LRP1	0	0	0	0	0	2	0	0	0	0	0	0	0	7	1	5	0	2	0	0	0	0	0	0	0	0	0	0	0	4	1	5	0	0	0	0	0	4	0	4	0	1	0	0
LRPAP1	0	2	0	0	1	0	2	0	0	0	0	0	0	0	1	0	2	0	1	1	0	0	0	0	0	0	0	0	0	2	0	1	0	0	3	0	0	7	0	5	1	0	0	0
MAP2	0	0	0	0	0	1	2	1	0	0	0	0	0	0	0	1	1	1	1	0	1	2	0	0	0	0	0	0	0	0	0	2	1	1	0	0	0	2	0	3	1	0	0	0
MARK4	0	0	0	1	0	2	0	4	0	0	0	0	1	1	4	0	0	0	0	1	1	0	0	0	0	0	0	0	0	1	0	3	0	2	0	0	1	5	2	1	0	0	0	0
MLL2	0	0	0	1	0	1	0	1	0	0	0	0	0	3	1	0	0	1	0	0	0	0	0	0	0	0	0	0	0	2	0	2	0	0	0	0	0	1	2	2	0	0	0	0
MMP15	0	0	1	0	1	1	7	0	0	0	0	0	1	0	0	0	0	2	0	0	0	0	0	0	0	0	0	0	1	1	0	1	0	1	0	0	1	1	2	0	0	0	0	0
MTCH2	0	0	0	0	0	2	0	4	0	0	0	0	2	0	1	0	0	3	0	3	0	0	0	0	0	0	0	0	0	1	1	0	1	1	0	0	0	5	0	2	0	1	0	0
NEDD9	0	0	0	0	0	3	0	3	0	0	0	0	0	0	2	0	1	1	0	0	0	0	0	0	0	0	0	0	0	1	0	3	0	0	0	1	0	14	0	19	0	6	0	2
PDC	0	2	0	0	0	10	0	7	0	0	0	0	0	1	0	2	0	2	0	4	0	0	0	0	0	0	0	0	0	5	0	11	0	2	0	0	0	7	0	6	0	0	0	1
PDIA3	0	0	0	0	0	0	0	0	0	0	0	0	1	0	1	0	0	0	0	0	1	0	0	0	0	0	0	0	4	0	2	0	0	0	0	1	4	1	7	1	0	0	0	1
PRKACA	0	0	0	0	0	2	0	5	0	0	0	0	0	1	0	1	0	1	0	2	0	0	0	0	0	0	0	0	1	0	1	0	1	1	0	3	0	49	0	19	0	0	0	4
PTPN7	0	0	0	0	0	17	0	8	0	0	0	0	0	6	0	2	0	2	0	2	0	0	0	0	0	0	0	0	0	5	0	11	0	1	0	0	0	8	0	9	0	2	0	1
RPS6KA1	3	1	0	0	1	0	0	0	0	0	0	0	0	0	0	0	0	0	0	0	2	0	0	0	0	0	0	0	0	1	1	2	0	0	0	0	3	5	0	0	0	0	2	0
RRAD	0	1	0	0	1	1	7	0	0	0	0	0	1	0	1	0	0	3	0	0	0	0	0	1	0	0	0	0	1	1	0	0	0	2	0	0	1	0	5	2	0	0	0	0
SMAD2	0	0	0	0	0	0	0	0	2	0	0	0	0	0	0	1	0	4	1	2	0	0	0	0	0	0	0	0	0	0	0	0	0	3	1	1	5	3	1	8	0	0	0	0
SNAP25	0	1	0	0	1	0	0	2	0	0	0	0	0	1	0	4	0	6	0	1	0	0	0	0	0	0	0	0	1	2	0	0	0	1	0	1	2	7	0	5	0	0	0	0
TH	0	0	0	0	0	0	1	1	0	2	0	2	1	0	7	0	2	0	0	0	0	0	0	0	0	0	0	0	0	0	0	0	0	0	0	0	1	1	1	1	1	1	0	0
TOP1	0	0	0	0	0	5	0	3	0	3	0	4	0	1	0	2	0	0	1	0	0	0	0	0	0	0	0	0	1	1	0	1	0	1	0	0	1	7	0	1	0	0	0	1

Supplementary Table 7. Drug targets of the genes in the multi-NCA biomarker.

The complete list of drug targets can be obtained from our web site http://bioinformatica.mty.itesm.mx/multicancer-biomarker.
